# Comprehensive Analysis of the Transcriptional and Mutational Landscape of Follicular and Papillary Thyroid Cancers

**DOI:** 10.1371/journal.pgen.1006239

**Published:** 2016-08-05

**Authors:** Seong-Keun Yoo, Seungbok Lee, Su-jin Kim, Hyeon-Gun Jee, Byoung-Ae Kim, Hyesun Cho, Young Shin Song, Sun Wook Cho, Jae-Kyung Won, Jong-Yeon Shin, Do Joon Park, Jong-Il Kim, Kyu Eun Lee, Young Joo Park, Jeong-Sun Seo

**Affiliations:** 1 Genomic Medicine Institute, Medical Research Center, Seoul National University, Seoul, Republic of Korea; 2 Interdisciplinary Program in Bioinformatics, Seoul National University, Seoul, Republic of Korea; 3 Department of Surgery, Seoul National University College of Medicine, Seoul, Republic of Korea; 4 Cancer Research Institute, Seoul National University College of Medicine, Seoul, Republic of Korea; 5 Research Institute, National Medical Center, Seoul, Republic of Korea; 6 Department of Biomedical Sciences, Seoul National University Graduate School, Seoul, Republic of Korea; 7 Department of Internal Medicine, Seoul National University College of Medicine, Seoul, Republic of Korea; 8 Department of Pathology, Seoul National University College of Medicine, Seoul, Republic of Korea; 9 Macrogen Inc., Seoul, Republic of Korea; 10 Department of Biochemistry and Molecular Biology, Seoul National University College of Medicine, Seoul, Republic of Korea; University of Michigan Medical School, UNITED STATES

## Abstract

Follicular thyroid carcinoma (FTC) and benign follicular adenoma (FA) are indistinguishable by preoperative diagnosis due to their similar histological features. Here we report the first RNA sequencing study of these tumors, with data for 30 minimally invasive FTCs (miFTCs) and 25 FAs. We also compared 77 classical papillary thyroid carcinomas (cPTCs) and 48 follicular variant of PTCs (FVPTCs) to observe the differences in their molecular properties. Mutations in *H/K/NRAS*, *DICER1*, *EIF1AX*, *IDH1*, *PTEN*, *SOS1*, and *SPOP* were identified in miFTC or FA. We identified a low frequency of fusion genes in miFTC (only one, *PAX8–PPARG*), but a high frequency of that in PTC (17.60%). The frequencies of *BRAF*^V600E^ and *H/K/NRAS* mutations were substantially different in miFTC and cPTC, and those of FVPTC were intermediate between miFTC and cPTC. Gene expression analysis demonstrated three molecular subtypes regardless of their histological features, including Non–*BRAF*–Non–*RAS* (NBNR), as well as *BRAF*–like and *RAS*–like. The novel molecular subtype, NBNR, was associated with *DICER1*, *EIF1AX*, *IDH1*, *PTEN*, *SOS1*, *SPOP*, and *PAX8–PPARG*. The transcriptome of miFTC or encapsulated FVPTC was indistinguishable from that of FA, providing a molecular explanation for the similarly indolent behavior of these tumors. We identified upregulation of genes that are related to mitochondrial biogenesis including *ESRRA* and *PPARGC1A* in oncocytic follicular thyroid neoplasm. Arm-level copy number variations were correlated to histological and molecular characteristics. These results expanded the current molecular understanding of thyroid cancer and may lead to new diagnostic and therapeutic approaches to the disease.

## Author Summary

Recently, The Cancer Genome Atlas proposed an improved classification of the subtypes of papillary thyroid carcinoma (PTC) based on gene expression profiles, which better represents cell signaling and differentiation. However, a molecular characterization of follicular thyroid carcinoma (FTC), which has a greater tendency for hematogenous spread to lung and bone is not yet fully elucidated. In this study, we describe the first RNA sequencing data of minimally invasive FTC (miFTC) and benign follicular adenoma (FA), which cause diagnostic difficulties due to their similar histological features. Additionally, classical PTC and follicular variant of PTC (FVPTC) were sequenced to compare their transcriptional and mutational landscape. *BRAF*, *H/K/NRAS*, fusion genes, and copy number variations were associated with tumor histology. Based on gene expression profiles, thyroid tumors were classified as three molecular subtypes regardless of histological subtypes, *BRAF*–like, *RAS*–like, and Non–*BRAF*–Non–*RAS*. In particular, we found identical gene expression profiles between miFTC, FA, and encapsulated FVPTC. Oncocytic follicular thyroid tumors have gene expression signatures related to mitochondrial biogenesis including *ESRRA* and *PPARGC1A*. These results expanded the current molecular understanding of thyroid cancer to its follicular types.

## Introduction

Most thyroid cancers are classified as either classical papillary thyroid carcinoma (cPTC), follicular variant of PTC (FVPTC), or follicular thyroid carcinoma (FTC) based on histological architecture [1]. However, the distinction between follicular-patterned thyroid tumors, such as FVPTC, FTC, and benign follicular adenoma (FA), still remains as a challenging problem [2]. Moreover, FTC and FA are indistinguishable by preoperative diagnosis as in practice they are often jointly referred to as follicular thyroid neoplasm (FTN) [3].

FTC accounts for approximately 10% of all thyroid cancers [4] and is known to harbor *H/K/NRAS* mutations, which are one of the molecular markers used for diagnosis [5]. However, *H/K/NRAS* mutations are also found in FVPTC and FA [6,7]. Therefore, these mutations are not sufficient as predictors of pure follicular histology or malignant potential in thyroid cancer.

The recent publication of The Cancer Genome Atlas (TCGA) studied molecular characteristics of PTC including the subtypes of classical type, tall cell variant, and follicular variant [8]. It was the first comprehensive pan-genomic study of thyroid cancer. They concluded that classification with two molecular subtypes, *BRAF*^V600E^–**like and *RAS*–like, represents the underlying signaling and differentiation properties better than pathological classifications. However, the analysis of TCGA was confined to subtypes of PTC and molecular characterization of FTC has not been performed.

In particular, the TCGA study demonstrated that the mitogen-activated protein kinase (MAPK) signaling pathway in PTC, as well as differentiation of thyroid cells, was differently regulated depending on molecular subtypes. There are some other reports about differential activation of the MAPK signaling pathway through several different genetic events such as *RET/PTC* fusions, *BRAF*, and *H/K/NRAS* point mutations [9,10]. The initiation of those genetic alterations likely depends on some triggering factor such as radiation or chemical elements [11–15]. However, the association between clinical risk factors and genetic alterations has not been fully understood yet.

We have performed a comprehensive RNA sequencing (RNA-seq) analysis to reveal the molecular characteristics of thyroid cancer including minimally invasive FTC (miFTC) and FA, and investigated their association to clinical data. Since there is no preceding large-scale RNA-seq study on miFTC and FA, we expect that our result will facilitate the discovery of new diagnostic and therapeutic approaches to thyroid cancer.

## Results

### Driver mutations of thyroid tumors

**Fig 1:**
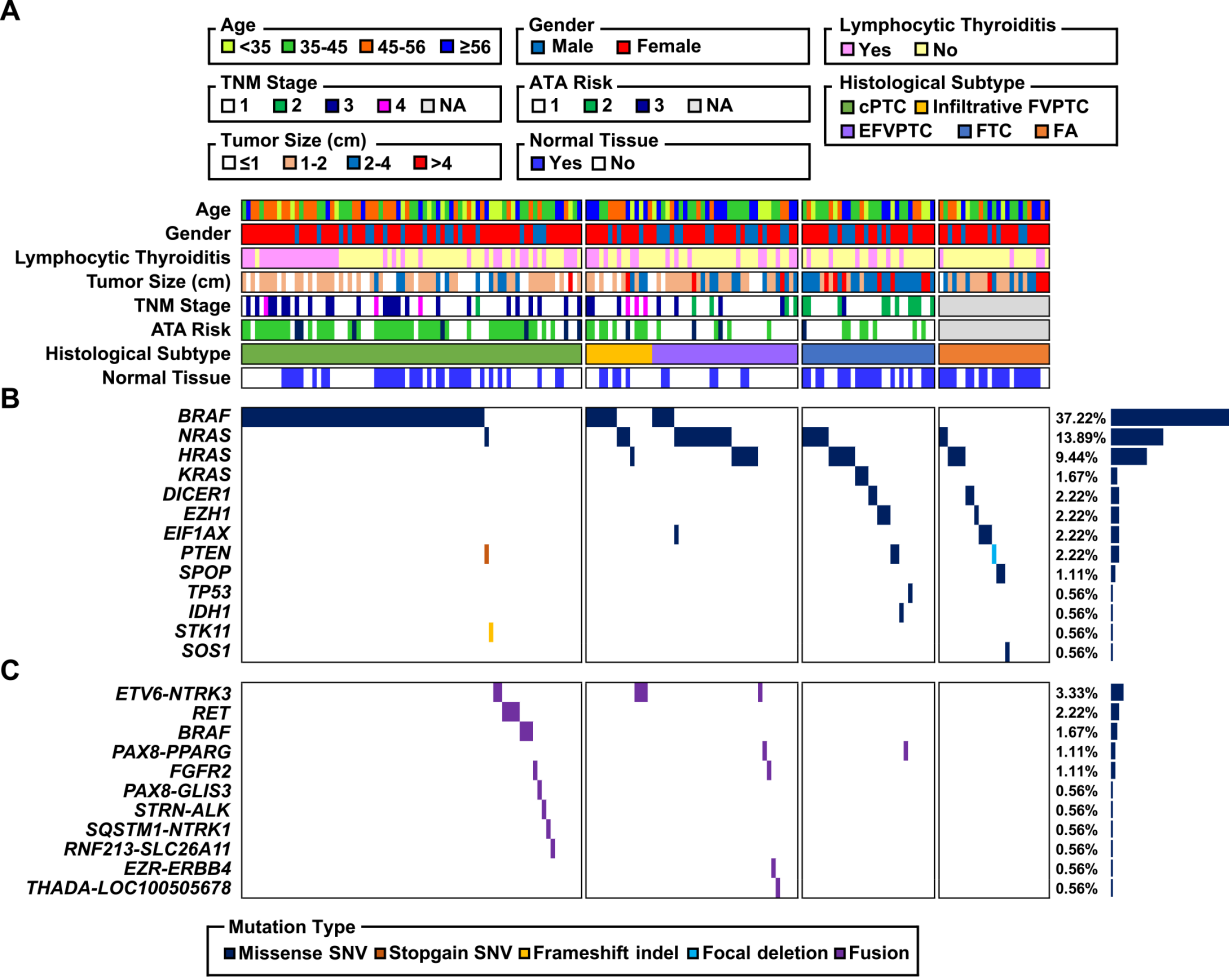
The mutational landscape of thyroid tumors. Each column represents an individual sample. (A) Age, gender, the presence of lymphocytic thyroiditis, tumor size, TNM Classification of Malignant Tumors stage, American Thyroid Association risk stratification, availability of matched normal tissue, and histological subtype. (B) Frequency of small size mutation by gene (right) and distribution of mutation across the 180 tumors (middle). (C) Frequency of fusion mutation by gene (right) and distribution of mutation across the 180 tumors (middle).

The mutational landscape of 180 thyroid tumors including 25 FAs, 30 miFTCs, 48 FVPTCs, and 77 cPTCs is illustrated in Fig 1. Mutations in well-known cancer driver genes (*BRAF* and *H/K/NRAS*) and fusion gene rearrangements were identified in 37.22%, 25.00%, and 12.78% of total tumors, respectively. The patterns of genetic alteration differed between PTC and FTN; most fusion genes were observed in PTC (17.60% and 1.82% in PTC and FTN, respectively; *p* = 0.002), while most mutations except *BRAF*^V600E^ and *H/K/NRAS* were found in FTN (32.73% and 0.80% in FTN and PTC, respectively; *p* < 0.0001). *BRAF*^V600E^ was only identified in PTC and its frequency differed between cPTC and FVPTC (71.43% and 25.00%, respectively; *p* < 0.0001). Many *H/K/NRAS* mutations were identified in FVPTC, miFTC, and FA (47.92%, 50.00%, and 24.00%, respectively). Only 1.30% of cPTC harbored *NRAS* mutations.

Four tumors (6.67% of miFTC and 8.00% of FA) harbored somatic *DICER1* mutations (E1705Q, D1810H, E1813G, and E1813Q; S1A Fig). These mutations were mutually exclusive with *BRAF*^V600E^ and *H/K/NRAS* mutations in FA as well as miFTC. The expression level of *DICER1* was increased with these somatic mutations (S1B Fig). Among these, two mutations were previously reported in TCGA study (D1810H and E1813G in TCGA-EL-A3GO and TCGA-EL-A3D5, respectively). They also tended to be mutually exclusive with *BRAF* and *H/K/NRAS* mutations (S1C Fig). Several mutations in the Ribonuclease III domain of *DICER1* were previously reported in PTC and other types of cancer [16–19], but *DICER1*^E1705Q^ mutation was first to be identified in thyroid tumor. We found three missense mutations in *EIF1AX* (G9V, R13C, and R13L) which was recently proposed as a driver gene in PTC (S1C Fig) [8]. These mutations were occurred more often in FA than in PTC (12.00% and 0.80%, respectively; *p* = 0.015) and they were mutually exclusive with *BRAF*^V600E^ and *H/K/NRAS* mutations. In addition, there were *IDH1*^R132C^ and two *PTEN* missense mutations (V343E and V175A) in miFTC. Also, one FA sample appeared to have a somatic focal deletion of *PTEN* based on its lacked expression in tumor. Furthermore, we suggest some novel driver candidates: *SOS1*^N233Y^, *SPOP*^P94R^, *EZH1*^Q571R^, *EZH1*^Y642F^, and *STK11*^R86fs^. *SOS1*^N233Y^ was identified as a recurrent hotspot in several cancers including uterine endometrial carcinoma, lung adenocarcinoma, and cancer cell lines [20]. *SPOP*^P94R^ was localized to the MATH domain and most somatic mutations in *SPOP* occurred in this domain [21,22]. *EZH1* is a member of the Polycomb group protein complex which are important components for prevention of cancer stem cell development [23]. In the TCGA dataset, *SPOP*^P94R^ and *EZH1*^Y642F^ tended to be mutually exclusive with *BRAF* and *H/K/NRAS* mutations (S1C Fig). Mutations in *STK11* were also reported in other types of cancer including poorly differentiated and anaplastic thyroid carcinoma [24,25]. Those mutations, *SPOP*^P94R^, *EZH1*^Q571R^, *DICER1*^E1813G^, *DICER1*^E1813Q^, and *EIF1AX*^R13C^, were confirmed as somatic mutations by polymerase chain reaction (PCR) and Sanger sequencing in tumor and matched normal tissues (S2A Fig).

We described all predicted fusion genes, breakpoint regions, and expression levels in S1 Table. All fusion genes including novel candidates were mutually exclusive with other mutations. Previously reported fusion genes in thyroid cancer, *ETV6–NTRK3* (4.80% in PTC), *CCDC6–RET* (2.40% in PTC), *NCOA4–RET* (0.80% in PTC), *SQSTM1–NTRK1* (0.80% in PTC), *STRN–ALK* (0.80% in PTC), and *PAX8–PPARG* (0.80% and 1.82% in PTC and FTN, respectively), were also identified [8]. *ALK*, *RET*, and *NTRK1* represented aberrant overexpression after fusion gene breakpoint (S3A Fig). Moreover, *ETV6–NTRK3* and *STRN–ALK* were validated by fluorescence *in situ* hybridization (FISH) and immunohistochemistry (IHC) staining, respectively (S3B and S3C Fig). In case of *BRAF*, we identified two novel candidate fusion genes, *PICALM–BRAF* (0.80% in PTC) and *NFYA–BRAF* (0.80% in PTC), in addition to formerly reported *SND1–BRAF* (0.80% in PTC) [8]. *PICALM–BRAF* was validated by reverse transcriptase PCR (RT-PCR) and Sanger sequencing (S2B Fig). Additionally, we suggest other fusion gene candidates such as *EZR–ERBB4*, *FGFR2–KIAA1598*, *FGFR2–WARS*, *PAX8–GLIS3*, *THADA–LOC100505678*, and *RNF213–SLC26A11*. From the above fusion gene candidates, *EZR–ERBB4*, *FGFR2–KIAA1598*, and *RNF213–SLC26A11* were identified in other types of cancer [26–28]. *ERBB4* also had aberrant overexpression after fusion gene breakpoint (S3A Fig). *THADA* rearrangement was previously reported in FA and PTC [8,29]. *PAX8* and *GLIS3* are both related to thyroid metabolism and function [8].

### Association between clinical risk factors and genetic alterations

Each subject showed different combinations of clinical risk factors such as age, smoking, alcohol drinking, menopausal status, and the presence of lymphocytic thyroiditis (LT). To investigate the association of these risk factors with genetic alterations, we categorized the patients into three groups: 1) small size mutation, 2) fusion gene, and 3) driver-unknown (Table 1). The average age of the fusion gene group (39.2 ± 13.1) was younger than driver-unknown (52.3 ± 14.9) and small size mutation (47.4 ± 12.1) groups (*p* = 0.002). Moreover, tumors with fusion gene were found more frequently in young adults (20.00% of subjects age < 45 yrs. and 6.26% of subjects age ≥ 45 yrs.; *p* = 0.006). The percentage of pre-menopausal women in the fusion gene group (75.00%) was higher than driver-unknown (23.53%) and small size mutation (55.00%) groups (*p* = 0.01). Patients harboring fusion gene were less likely to smoke than others, but it was not statistically significant. Also, the percentage of patients who drink alcohol was not different among the groups. Patients harboring *H/K/RAS* mutations had a lower frequency of LT (11.11%), which was defined by histologic findings in normal thyroid parenchyma, than *BRAF*^V600E^ (37.31%; *p* = 0.002) and fusion gene (47.83%; *p* = 0.0004) groups. The fusion gene group showed higher frequency of LT compared with driver-unknown (26.92%) and other mutation (21.05%) groups, although this was not statistically significant.

**Table 1:** Comparison of clinical risk factors among the groups with different types of mutation.

Variable	Driver-unknown	Small size mutation				Fusion gene	*p*-value ^a^	*p*-value ^b^
		Total	*BRAF*	*H/K/NRAS*	Others			
N	26	131	67	45	19	23		
Age	52.3 ± 14.9^c^	47.3 ± 12.1^c^	47.2 ± 12.0	48.1 ± 12.5	45.8 ± 12.1	39.2 ± 13.1	**0.002**	**0.010**
Sex (female)	21 (80.77)	93 (70.99)	50 (74.62)	28 (62.22)	15 (78.95)	17 (73.91)	0.587	0.425
Pre-menopause (regular)	4/17 (23.53)^c^	44/80 (55.00)	20/38 (52.63)	14/27 (51.85)	10/15 (66.67)	12/16 (75.00)	**0.010**	**0.038**
Smoking (current + ex)	3 (11.54)	20/130 (15.38)	9 (13.43)	7/44 (15.91)	4 (21.05)	1 (4.35)	0.431	0.578
Male	1/5 (20.00)	16/38 (42.11)	8/17 (47.05)	6/17 (35.29)	2/4 (50.00)	1/6 (16.66)	0.446	0.659
Female	2/21 (9.52)	4/92 (4.35)	1/50 (2.00)	1/27 (3.70)	2/15 (13.33)	0/17 (0.00)	0.400	0.172
Drinking (current)	7 (26.92)	40/130 (30.77)	16 (23.88)	16/44 (36.36)	8 (42.11)	7 (30.43)	0.926	0.482
Lymphocytic thyroiditis	7 (26.92)	34 (25.95)	25 (37.31)	5 (11.11)^c^^,^^d^	4 (21.05)	11 (47.83)	0.100	**0.008**

^a^ *p*-value for comparison among Driver-unknown, Total, and Fusion gene mutation groups.

^b^ *p*-value for comparison among Driver-unknown, *BRAF*, *H/K/NRAS*, Others, and Fusion gene mutation groups.

^c^ Significantly different from “Fusion gene mutation” group. (*p* < 0.05 for post-hoc Bonferroni test)

^d^ Significantly different from “*BRAF*” group. (*p* = 0.002 for post-hoc Bonferroni test)

### Gene expression analysis on thyroid tumors

**Fig 2:**
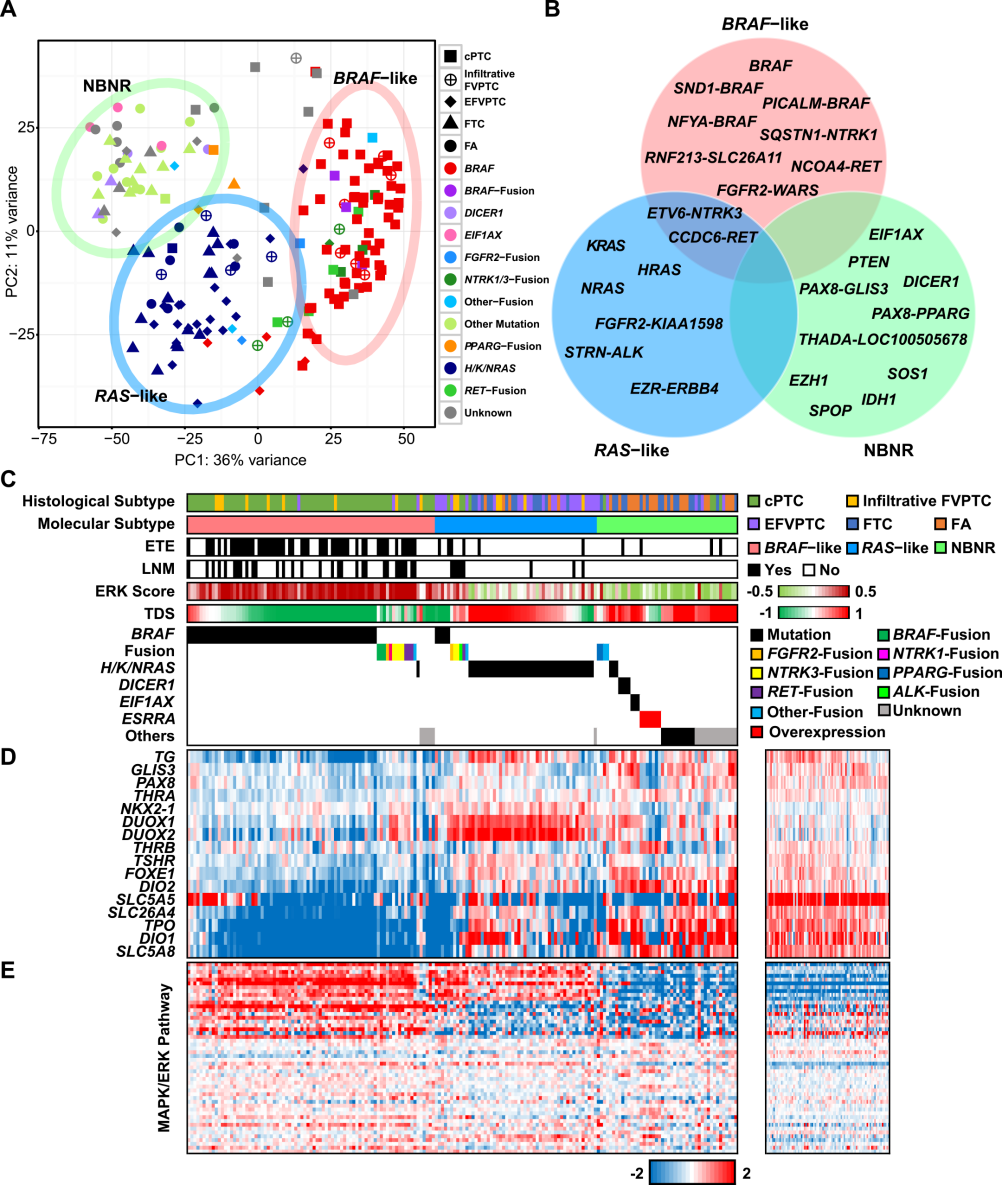
Gene expression analysis on thyroid tumors. (A) The result of K-means clustering via PCA. Three distinct molecular subtypes were found: *BRAF*–like, *RAS*–like, and NBNR. Each cluster was represented by a 95.00% confidence ellipse. (B) Driver gene of three molecular subtypes. (C) Histological subtype, molecular subtype, the presence of extrathyroidal extension, the presence of lymph node metastasis, ERK score, TDS, and driver gene in individual samples. 180 tumors were sorted by molecular subtype, driver gene, and high to low TDS. (D) The heat maps represent expression level of 16 thyroid metabolism and function genes and (E) the MAPK signaling pathway genes in tumor (middle) and normal (right) specimens. Genes were clustered by K-means clustering algorithm (K = 3).

The result of K-means clustering via principal component analysis (PCA) on all study subjects is shown in S4A Fig. Tumor and normal tissues were distinctively separated in the PC2 axis even though some of them were grouped together in one of the K-means cluster. This cluster was associated with LT which was observed in 28.89% of study subjects (S4B Fig). Samples with *BRAF*^V600E^ mutation and LT were also separated from samples with *BRAF*^V600E^ mutation and without LT when the same analysis was conducted with only tumors (S4C and S4D Fig). In case of TCGA dataset, we were not able to distinguish an LT derived cluster although 22.89% of specimens harbored LT (S4E Fig). The inconsistent result between TCGA and the current study could be raised from different gene set usage for each analysis; we used the Ensembl gene set instead of the UCSC gene set which was used in TCGA study. Within the most variable 500 genes in the Ensembl gene set applied to PCA, 91 genes were associated with immunoglobulin and only four genes were overlapped with the UCSC gene set. In order to decrease the gene expression variation affected by LT and increase that derived from oncogenic signal transduction, we used genes covered by the UCSC gene set for molecular classification. With this approach, we obtained three molecular subtypes in relation to oncogenic signal transduction: *BRAF*–like, *RAS*–like, which were proposed by TCGA, and a third which we refer to as Non–*BRAF*–Non–*RAS* (NBNR). The three molecular subtypes that we identified showed a clear separation of samples by driver genes (Fig 2A). We could get exceedingly similar result when the same analysis was performed on TCGA dataset (S4F Fig). As the effect of gene expression derived by *BRAF* and *H/K/NRAS* was overwhelming in PCA due to their huge sample size, this analysis was performed on a partial TCGA dataset.

*BRAF*–like consisted of *BRAF*^V600E^ and fusion genes (*PICALM–BRAF*, *NFYA–BRAF*, *SND1–BRAF*, *FGFR2–WARS*, *ETV6–NTRK3*, *SQSTM1–NTRK1*, *CCDC6–RET*, *NCOA4–RET*, and *RNF213–SLC26A11*). None of FTN was clustered into *BRAF*–like because of their skewed proportion of *BRAF*^V600E^ and fusion genes. *RAS*–like consisted of *H/K/NRAS* and fusion genes (*STRN–ALK*, *EZR–ERRB4*, *FGFR2–KIAA1598*, *ETV6–NTRK3*, and *CCDC6–RET*). Lastly, NBNR was associated with *DICER1*, *EIF1AX*, *IDH1*, *PTEN*, *PAX8–PPARG*, and other driver gene candidates (Fig 2B).

The aggressive pathologic characteristics, lymph node metastasis (LNM) and extrathyroidal extension (ETE) were correlated with the 3 molecular subtypes (Fig 2C); higher frequency of LNM (37.04%) or ETE (61.73%) was found in the *BRAF*–like group, while less or no LNM or ETE was observed in the *RAS*–like group (15.09% of LNM, 11.32% of ETE) or NBNR group (0.00% of LNM, 8.70% of ETE) (For both categories; *p* < 0.0001).

To measure differentiation of thyroid cells and activation of the MAPK signaling pathway in three molecular subtypes, we implemented two scoring methods that were introduced by TCGA study: thyroid differentiation score (TDS) and ERK score (Fig 2C) [8]. Most *BRAF*–like tumors had low TDS, while *RAS*–like and NBNR tumors had high TDS. There was a strong negative correlation between TDS and molecular subtype classification (Pearson correlation coefficient = -0.66). The low level of TDS was derived from decreased expression level of 16 thyroid metabolism and function genes [8]. Many of these 16 genes were downregulated in *BRAF*–like, while *RAS*–like and NBNR maintained stable gene expression levels. In *BRAF*–like, significantly downregulated genes were *DIO1*, *DIO2*, *TPO*, *SLC26A4*, and *SLC5A8*. *DUOX1* and *DUOX2* were increased in *RAS*–like. On the other hand, NBNR had no differentially regulated gene except *ESRRA* overexpressed tumors (See “The characteristic gene expression of oncocytic FTN” section), which represented downregulation of several genes: *DIO1*, *FOXE1*, *GLIS3*, *PAX8*, and *SLC5A5* (Fig 2D).

The involvement of constitutive activation of the MAPK signaling pathway in the pathogenesis of PTC is well established [9]. ERK score strongly represented activation level of MAPK signaling pathway and there was very strong positive correlation between ERK score and molecular subtype classification (Pearson correlation coefficient = 0.80). As discussed in TCGA study, ERK score was highly elevated in most *BRAF*–like, but not in *RAS*–like samples. Although *RAS*–like represented lower ERK score than *BRAF*–like, it had some activated genes in the MAPK signaling pathway. However, NBNR did not have activated genes as represented by the ERK score (Fig 2E).

### Gene expression analysis on follicular-patterned thyroid tumors

The mutational profile of miFTC and FA were very similar to each other. Moreover, that of EFVPTC was also similar to FTNs, while that of infiltrative FVPTC was similar to cPTC (Fig 1). All these tumors are follicular-patterned, which are occasionally hard to distinguish from one another. To identify the transcriptional difference among these follicular-patterned thyroid tumors, we performed PCA and differentially expressed gene (DEG) analysis.

**Fig 3:**
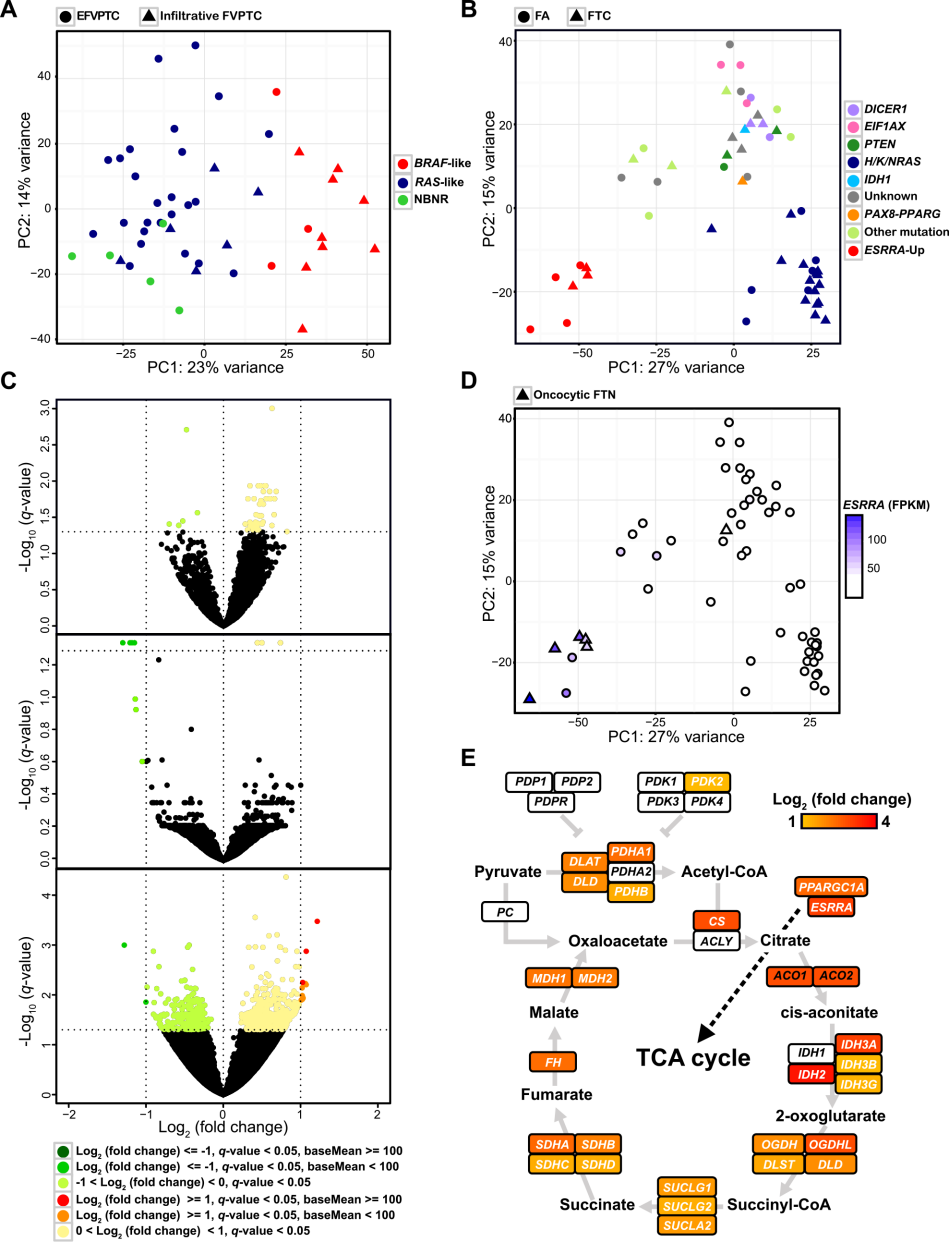
Gene expression analysis on follicular-patterned thyroid tumors. (A) The result of PCA on FVPTC. (B) The result of PCA on miFTC and FA. (C) The volcano plots represent identical gene expression among three subtypes: miFTC and FA (upper), FA and EFVPTC (middle), and miFTC and EFVPTC (lower). All analysis was restricted to *RAS*–like tumors. (D) *ESRRA* expression level of miFTC and FA. (E) The characteristic gene expression of oncocytic FTN. *ESRRA*, *PPARGC1A*, genes in TCA cycle were represented by Log_2_ (fold change). The illustration was generated based on a figure from Davis et al and KEGG pathway [34,54].

In PCA performed on EFVPTC and infiltrative FVPTC, PC1 axis clearly divided tumors which were classified as *BRAF*–like and *RAS*–like/NBNR (Fig 3A). EFVPTC was mainly associated with *RAS*–like/NBNR rather than infiltrative FVPTC (*p* = 0.0004). When we performed PCA on miFTC and FA which are hard to distinguish by pathological examination, we could not find any cluster nor PC axis that separates miFTC and FA. Several clusters and PC axes divided those tumors, but all groups consisted of miFTC and FA. (Fig 3B). The lower right corner and the upper central group were associated with *H/K/NRAS* and other driver genes (e.g., *DICER1*, *EIF1AX*, *IDH1*, *PTEN*, and *PAX8–PPARG*), respectively. DEG analysis also confirmed that miFTC and FA did not have significant transcriptional difference. Moreover, the transcriptome of EFVPTC which shows indolent behavior was also indistinguishable from miFTC and FA (Fig 3C).

### The characteristic gene expression of oncocytic FTN

We identified the increased *ESRRA* expression level of tumors in the lower left corner group in PCA performed on miFTC and FA (Fig 3B and 3D). Pathway enrichment analysis [30] on chemical and genetic perturbations database showed that DEGs of the aforementioned cluster harbored genes that were upregulated by *ESRRA* and were related to mitochondria (S2 Table). Remarkably, most of those tumors were oncocytic FTN (*p* < 0.0001); 83.33% of oncocytic FTN (five out of six) was clustered into *ESRRA* overexpression group (Fig 3D).

Oncocytic FTN is characterized by remarkable accumulation of mitochondria [31]. In those tumors, expression level of *ESRRA* showed very strong positive correlation with expression level of *PPARGC1A* (Pearson correlation coefficient = 0.83 using FPKM). Both *ESRRA* and *PPARGC1A* are key regulators of mitochondrial biogenesis [32,33]. DEG analysis demonstrated that the majority of genes in citric acid cycle (TCA cycle) were dramatically upregulated in oncocytic FTN (Fig 3D). All of the oncocytic FTNs were classified as NBNR (Fig 2C).

### Pathway analysis on molecular subtypes

To investigate detailed gene expression signatures in the three molecular subtypes, we performed pathway enrichment analysis on DEGs of each molecular subtype using the Kyoto Encyclopedia of Genes and Genomes (KEGG) pathway database [34]. The top 20 most significantly enriched KEGG pathways of upregulated and downregulated genes of each molecular subtype are illustrated in S5 Fig and S6 Fig, respectively. In *BRAF*–like, pathways including cell adhesion molecules (CAMs), the extracellular matrix (ECM) receptor interaction, and focal adhesion were remarkably upregulated. The involvement of these pathways in the carcinogenesis of thyroid nodules and cancer invasiveness had been reported previously [35–37]. Moreover, the risk assessments based on TNM Classification of Malignant Tumors (TNM) stage and American Thyroid Association (ATA) risk stratification supported that *BRAF*–like is more aggressive than other molecular subtypes (*p* = 0.030 and *p* = 0.001, respectively). The p53 and MAPK signaling pathways were upregulated in both *BRAF*–like and *RAS*–like but not in most of NBNR. Numerous metabolism and calcium signaling pathways were downregulated in *BRAF*–like, and these pathways were barely downregulated in other molecular subtypes.

Novel molecular subtype NBNR is composed of diverse kinds of driver genes and they had different gene expression profiles depending on the types of mutated or overexpressed gene. As we mentioned earlier, upregulated genes of *ESRRA* overexpressed tumors were significantly enriched for pathways related to TCA cycle, oxidative phosphorylation (OXPHOS), PPAR signaling, and several metabolisms. Moreover, tumors with *PAX8*–*PPARG* rearrangement also showed increased metabolism and PPAR signaling pathways. The Wnt and mTOR signaling pathways were enriched in *DICER1* and *EIF1AX* mutated tumors, respectively.

### Copy number variation in thyroid tumors

**Fig 4:**
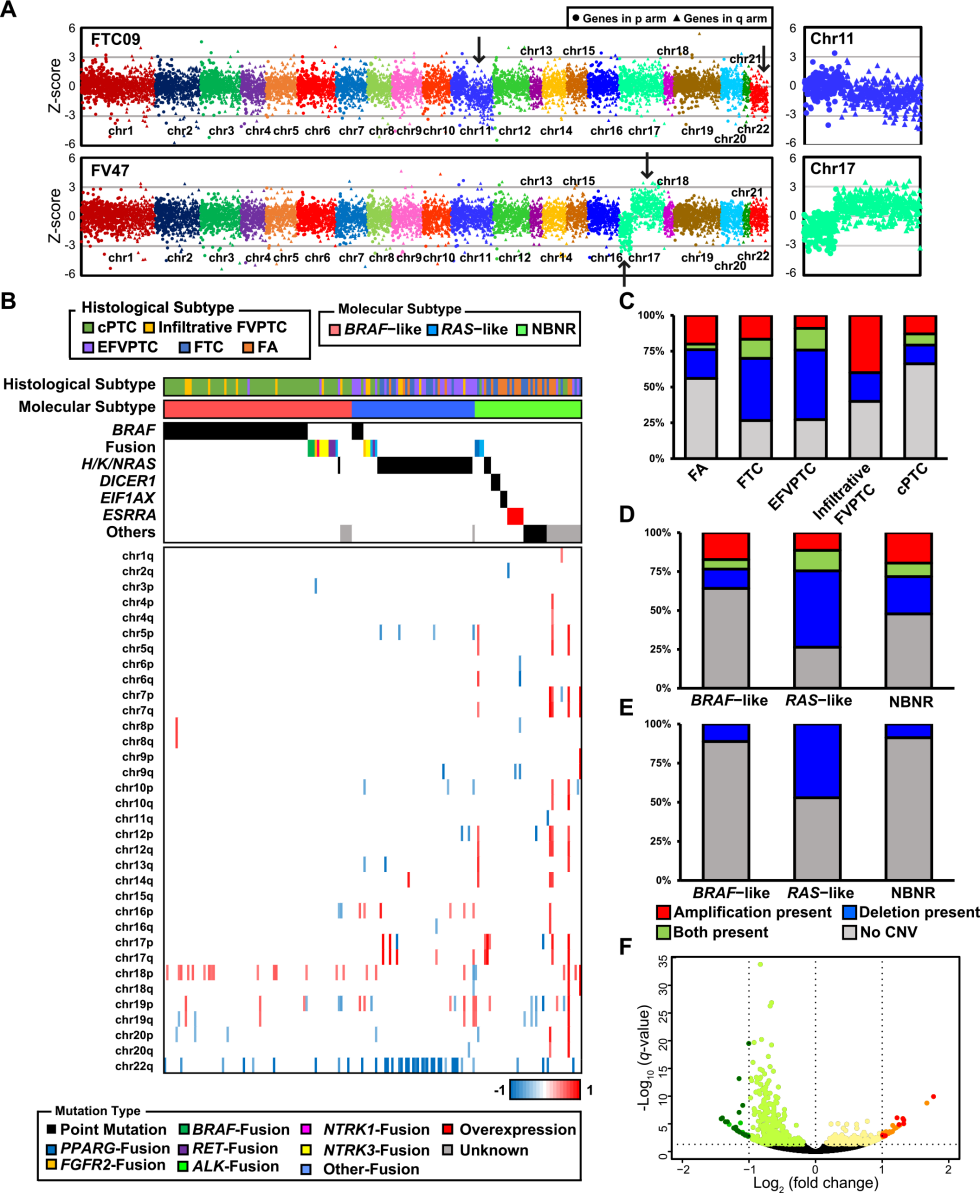
The CNV landscape of thyroid tumors. (A) The examples of arm-level duplication and deletion were pointed by arrow. Enlarged images of chromosome 11q deletion (upper right) and chromosome 17 with deletion and duplication in p and q arm (lower right) were provided. (B) The distribution of CNVs across 180 tumors. The chromosome arms without CNV across all tumors were excluded. Specimens were sorted in the same manner as Fig 2. (C) The percentage of CNV in each histological subtype. (D) The percentage of CNV in each molecular subtype. (E) The percentage of chromosome 22q deletion in each molecular subtype. (F) The volcano plot shows massive number of moderately downregulated genes in *RAS*–like tumors with chromosome 22q deletion. Positional gene set enrichment result of these genes is provided in S4 Table.

To identify arm-level copy number variations (CNVs) of thyroid tumors, we defined arm-level jointly regulated blocks (JRBs) which demonstrate colocalization of overexpressed and underexpressed chromosome arms. Our previous study demonstrated high correlation between JRB and CNV status in cancer genomes [38]. In this study, we modified our former method to define arm-level JRBs. We successfully predicted aberration of chromosome arms which represent arm-level amplification and deletion (Fig 4A). We illustrated the CNV landscape of all thyroid tumors in Fig 4B. cPTC had the lowest percentage of arm-level CNV, while miFTC, EFVPTC, and infiltrative FVPTC had high percentage of that. FA showed a lower percentage of arm-level CNV than miFTC (Fig 4C). It was reported that chromosome 12 is more frequently amplified in FA and indolent tumors than aggressive tumors [39]. We also identified amplification of chromosome 12 in FTN but not in PTCs (*p* = 0.008). The percentage of arm-level deletion was higher in *RAS*–like than *BRAF*–like and NBNR (Fig 4D). Similar to previous reports [8,39], deletion of chromosome 22q was the most frequently identified arm-level CNV in *RAS*–like (Fig 4E; *p* < 0.0001). DEG test on *RAS*–like tumors with or without chromosome 22q deletion confirmed the reliability of detecting CNV by our approach. The moderately downregulated genes (-1 < Log_2_ (fold change) < 0, *q*-value < 0.05) were enriched to chromosome 22q when positional gene set enrichment was performed (Fig 4F and S3 Table). However, there was no difference in other clinicopathological features whether chromosome 22q deletion occurred in *RAS*–like or not; only multifocality was more significantly frequent (S4 Table; *p* = 0.037). In *BRAF*–like, amplification of chromosome 18p was more frequent than other molecular subtypes, but it was not statistically significant (*p* = 0.051). The ratio of LT was elevated when chromosome 18p was amplified (S5 Table; *p* = 0.032,).

## Discussion

Recently, the genomic landscape of PTC has been well investigated [8]. This study reduced the rate of unknown oncogenic drivers in subtypes of PTC from 25% to 3.5% through discoveries of somatic alterations including *EIFIAX*, *PPM1D*, *CHEK2*, and diverse fusion genes. However, the transcriptional and mutational landscape of miFTC, which has a greater tendency for hematogenous spread to lung and bone, is yet to be widely explored. In the present study, we performed RNA-seq on miFTC and FA, in addition to cPTC and FVPTC.

**Fig 5:**
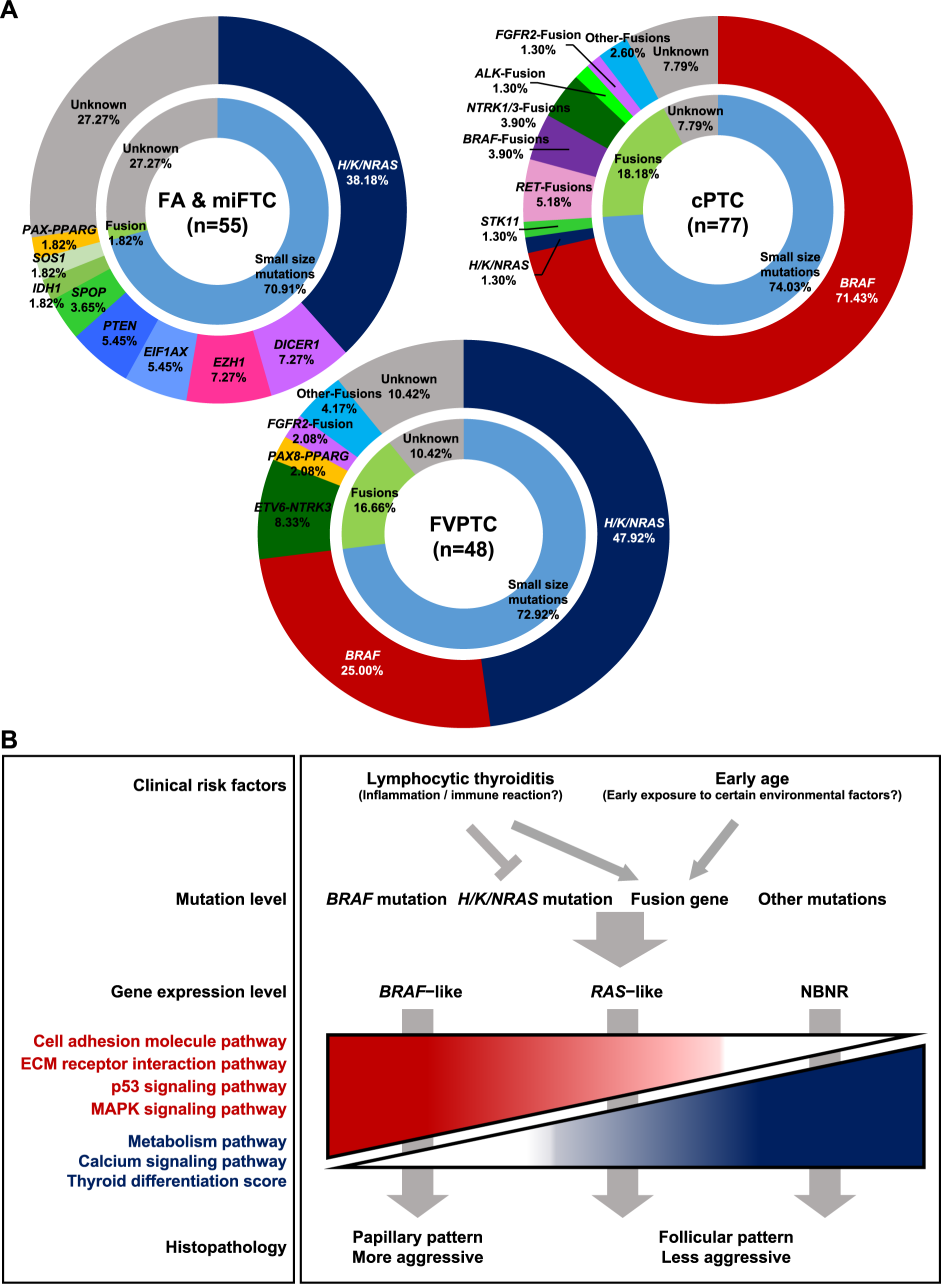
The overview of the present study. (A) The driver mutations in thyroid tumors. Each pie chart represents the distribution of driver mutations identified in FTN, FVPTC, and cPTC, respectively. (B) A schematic model of thyroid cancer progression integrating clinical risk factors, mutational, transcriptional profile, and clinical presentation. Tumor aggressiveness was determined by the presence of invasive pathologic characteristics of ETE or LNM.

We identified driver genes in 72.73%, 89.58%, and 92.21% of FTN, FVPTC, and cPTC samples, respectively (Fig 5A). The patterns of genetic alteration differed between histological subtypes. cPTC and miFTC showed considerably different patterns of genetic alteration to each other. However, FVPTC represented an intermediate mutational status between cPTC and miFTC; EFVPTC and infiltrative FVPTC were similar to miFTC and cPTC, respectively. Furthermore, miFTC and FVPTC have higher percentages of arm-level CNVs than cPTC (Fig 4C). This is consistent with previous studies that described a higher fraction of somatic copy number alterations in FVPTC and FTN than cPTC [8,40]. Taken together, our result suggests that different genetic alterations could lead to different tumor histology. In addition, we found that FA has a lower percentage of arm-level CNVs than miFTC. This result supports the hypothesis that FA is a preneoplastic condition of miFTC despite the similar patterns of genetic alteration between them.

It has been suggested that several clinical risk factors including smoking [41], alcohol drinking [42], LT [43,44], menopausal status [45], genetic predisposition [46], and early exposure to radiation [11,13,14] affect the development of thyroid cancer. However, there are few studies considering genetic alterations and clinical risk factors at the same time [12]. Therefore, we analyzed the possible association between types of genetic alteration and clinical risk factors to investigate the etiology of thyroid cancer (Table 1). The recent reports from Chernobyl cohort well demonstrated a relationship between fusion gene and thyroid cancer. [47,48]. Although there was no history of radiation exposure in our subjects, the younger age of the fusion gene group than other groups may reflects the involvement of environmental or genetic factors to the development of chromosomal rearrangement inducing thyroid cancer. Hashimoto’s thyroiditis is the main etiology of LT which is related to inflammation and immune reactions observed in thyroid. In this study, an elevated tendency of LT in the fusion gene group was shown and negative association between LT and *H/K/NRAS* mutation was also identified. These results suggest an etiologic role of LT in thyroid cancer development. Based on our findings, we suggest that some risk factors influence the types of genetic alteration. We believe that further study would allow better understanding of thyroid cancer development.

Based on transcriptional landscape, 180 tumors were classified as *BRAF*–like, *RAS*–like, and NBNR (Fig 2A). Our result in PTCs has similar context to the TCGA study, which classified subtypes of PTC as *BRAF*^V600E^–**like and *RAS*–like. It was reported that FVFTCs in TCGA, which are classified as *RAS*–like, were often misdiagnosed as FTC by pathologists [49]. Moreover, as we mentioned earlier, arm-level copy number alterations were frequently observed in FVPTC similarly to FTC as well as *H/K/NRAS* mutations [40]. The aforementioned issues raised a question regarding the distinction between FTC and FVPTC in the point of biological and clinical relevance. In our analysis, EFVPTC and infiltrative FVPTC showed different mutational and transcriptional characteristics to each other (Fig 1 and 3A). EFVPTC, which was recently re-classified as “noninvasive follicular thyroid neoplasm with papillary-like nuclear features” according to its indolent features [50] had highly similar gene expression profiles to miFTC or FA, (Fig 3C). This result emphasizes that re-classification of thyroid cancers based on their mutational and transcriptional characteristics may be beneficial for stratified medicine.

One of the goals of this study was to discover molecular markers to distinguish miFTC and FA. Differential diagnosis for FTC and FA is important for decisions to undergo surgery in clinic, but it is almost impossible due to their highly similar cytological features at present [2]. Several researchers have suggested markers based on gene expression levels [51–53], but they are not widely adopted. In our analysis, we could not find any significant transcriptional difference between miFTC and FA (Fig 3B and 3C). These results again suggest that miFTC is indolent and it could be treated minimally. However, the transcriptional difference between widely invasive FTC (wiFTC) and miFTC/FA is yet to be investigated as there was no wiFTC in the current study.

Most tumors harboring *EIF1AX* mutations and *PAX8–PPARG* rearrangement were classified as *RAS*–like in TCGA study. However, they were distinguished from *RAS*–like and were classified as NBNR according to current and TCGA datasets (Fig 2A and S4F Fig). Traditionally, thyroid cancer is well known to be associated with activation of the MAPK signaling pathway [9,10]. Our results suggested that NBNR involves totally different mechanism and pathways (Fig 2E). Furthermore, NBNR exhibited distinct gene expression profiles within the class (S5 Fig). We believe that accumulating data would lead to more effective molecular classification and to discovery of therapeutic targets.

In *BRAF*–like, higher activation of ECM receptor interaction, CAMs, p53, and MAPK signaling pathways than other molecular subtypes was identified (S5 Fig). Furthermore, low level of TDS and downregulation of several metabolism pathways supported poor clinical presentation in *BRAF*–like (Fig 2C and S6 Fig). We could not establish the clinical impact of molecular subtypes on locoregional recurrence (n = 1), distant metastasis (n = 4), and cancer-specific mortality (n = 0) due to the short median follow-up of 37 months (1–100 months) and low percentage of advanced thyroid cancer. However, the other aggressive pathologic characteristics LNM and ETE were observed much more frequently in the *BRAF*–like group (Fig 2C), demonstrating its association with clinical presentation or aggressiveness.

Collectively, we propose a schematic model of thyroid cancer progression integrating clinical risk factors, mutational and transcriptional landscape, and clinical presentation (Fig 5B).

The underlying mechanism of mitochondria accumulation in oncocytic FTN has not been elucidated clearly. We deduced that oncocytic FTN had distinct transcriptome among thyroid tumors containing extremely upregulated mitochondria-related metabolic pathways (Fig 3D and 3E, and S5 Fig). This feature was in agreement with a recent study on eosinophilic chromophobe renal cell carcinoma which is also characterized by densely packed mitochondria [54]. The stimulation of mitochondrial biogenesis and OXPHOS by *ESRRA* and *PPARGC1A* is well established [32,33] and upregulation of two genes supported mitochondria accumulation in oncocytic FTN. The stimulation of high expression level of *ESRRA* and *PPARGC1A* is not fully studied here. However, we believe that our findings could provide important clues to understand the role of mitochondrial biogenesis in oncocytoma. Recently, there was a study that suggested mechanism of oncocytic thyroid tumor development [55]. They demonstrated that many oncocytic thyroid tumors harbored copy number gained mitochondrial biogenesis genes including *ESRRA*.

In summary, this study demonstrates the transcriptional and mutational landscape of miFTC and FA together with cPTC and FVPTC. We revealed that thyroid cancers developed by different types of genetic alteration could be classified as three molecular subtypes (*BRAF*–like, *RAS*–like, and NBNR) based on gene expression profiles. The three molecular subtypes showed difference in chromosomal aberration, cell proliferation, differentiation, intracellular signaling, and metabolism. We propose that reclassification of thyroid tumors, especially follicular-patterned ones, on the basis of molecular characteristics would provide novel diagnostic implications.

## Materials and Methods

### Ethics statement

This study was approved by the institutional review board of Seoul National University Hospital, in accordance with the Declaration of Helsinki (approved ID: H-1108-041-372). Written informed consent was obtained from each subject.

### Patients

Specimens from 180 patients (49 men and 131 women; 47 ± 13 years of age) whose fresh frozen thyroid tissue after thyroid surgery were collected from March 2007 to January 2014. We could collect 180 tumor tissue samples (25 FAs, 30 FTCs, 48 FVPTCs, and 77 cPTCs) and 81 paired-normal tissue samples that matched with their tumor tissues. The diagnosis of each sample was determined based on pathological findings from thyroid specimens obtained after thyroidectomy. The clinical information of study subjects is shown in S6 Table. There were no patients who were exposed radiation previously.

### Pathological diagnosis

Pathological slides were reviewed by a specialized pathologist. cPTC was defined if the tumor has well-formed papillae with fibrovascular cores and characteristic nuclear features of papillary carcinoma. FVPTC was defined as a PTC with predominantly a follicular growth pattern more than 50%, no well-formed papillae. There are two subtypes of FVPTC: infiltrative FVPTC and EFVPTC regarding the tumor border—infiltrative border or a pushing border with smooth outlines and a capsule, respectively. Capsular invasion was identified in only two cases in EFVPTC and there was not capsular invasion in the other FVPTCs. Therefore, we did not categorize encapsulated FVPTC into two subgroups regarding capsular invasion. miFTC was diagnosed if the tumor is encapsulated by capsular invasion and/or small-caliber sized angioinvasion. FA was diagnosed with no capsular invasion and angioinvasion [56].

### RNA sequencing

Extraction of RNA from frozen tissues was performed using the QIAcube and RNeasy Mini Kit (Qiagen, Hilden, Germany) or the Easy Spin RNA extraction kit (Intron, Daejeon, Korea) when tissue volume was small but high product yield was needed. RNA was assessed for quality and concentration measurement using an RNA 6000 Nano LabChip on a 2100 Bioanalyzer (Agilent Inc., Palo Alto, CA). The sequencing libraries were sequenced on a HiSeq 2000 platform (Illumina, San Diego, CA).

The sequenced paired-end reads were aligned to GRCh37.p13 human reference genome using STAR 2-pass method [57,58] and PCR duplicates were removed by Picard MarkDuplicate (http://picard.sourceforge.net). Filtered reads were further processed for variant calling using best-practice of GATK (https://software.broadinstitute.org/gatk/best-practices/), which includes insertion/deletion (indel) realignment and base quality score recalibration [59]. S8 Table shows a summary of sequencing throughput and alignment yield in our study subjects.

### SNV and indel calling

We called somatic single-nucleotide variants (SNVs) from 81 matched samples using MuTect [60]. For non-matched samples, we applied SNV calling using the single sample mode of MuTect and GATK’s HaplotypeCaller. Moreover, GATK’s HaplotypeCaller was also used for indel detection. All variants called in these manners were annotated with information from several databases using ANNOVAR [61]. Furthermore, we used GATK’s DepthOfCoverage for counting alternative allele of mutation hotspots in common oncogenes.

To discover driver mutations in thyroid cancers, we applied additional filtration criteria to variant calls, as follows: 1) not or rarely shown in public databases of normal individuals, such as Exome Aggregation Consortium (ExAC) (http://exac.broadinstitute.org/), 1000 Genomes projects [62] and Exome Sequencing Project 6500 (http://evs.gs.washington.edu/EVS/) (MAF ≤ 0.0001 for ExAC and ≤ 0.01 for other databases); 2) nonsilent SNVs (nonsynonymous and splice-site) and frameshift indels; 3) genes that were annotated in COSMIC70 or PTC dataset of TCGA project. Driver candidates in TCGA were examined using cBioPortal for Cancer Genomics [63].

### Fusion gene mutation analysis

To discover fusion genes in thyroid cancers, we used MOJO (https://github.com/cband/MOJO) with TCGA GAF 3.0 reference. For filtering false positive calls, we applied further filtration to predicted calls: 1) fusion genes only shown in tumor samples; 2) discordant read pairs between gene A and B ≥ 2; and 3) genomic distance between predicted coordinates ≥ 100 kb or two genes located on different chromosomes.

### Validation of novel driver candidates

To validate the mutation of novel driver candidates, gDNA was extracted using QIAamp DNA Kits and QIAamp DNA FFPE Tissue Kit (Qiagen, Hilden, Germany) from fresh-frozen tissues, or formalin-fixed, paraffin-embedded (FFPE) tissue specimens. DNA was quantified using a Nanodrop ND-1000 Spectrophotometer (NanoDrop Technologies Inc., Wilmington, USA) and used as template for PCR amplification. Amplification primers were designed with Primer3 [64]. PCR was performed on GeneAmp PCR System 9700 (Applied Biosystems; Life Technologies, Carlsbad, USA) using Hotstar Taq polymerase (Qiagen) as follows; 15 minutes at 95°C for initial denaturation, then 40 cycles at 95°C for 30 seconds, 60°C for 30 seconds, and 72°C for 60 seconds, then 5 minutes at 72°C for final extension. 20 ng of gDNA was used to amplify. Amplified products were purified with DNA purification kit (HiYield DNA fragment extraction kit, Real Genomics, New Taipei City, Taiwan), and then analyzed on an Applied Biosystems 3730XL DNA Sequencing Facility (Applied Biosystems). PCR primer and DNA sequencing services were provided by Cosmo Genetech (Cosmo Genetech, Seoul, Korea). The sequencing results were analyzed using ABI Sequencing analysis software 5.2 (Applied Biosystems). The primer sequences used in this study provided in S8 Table.

### Validation of novel fusion gene candidates

RT-PCR and Sanger sequencing were performed across the fusion break points to identify the exact fusion junction of *PICALM–BRAF*. The tissue blocks were cut into 4μm slides, and total RNA from FFPE samples was isolated using a tissue kit (Maxwell 16 LEV RNA FFPE purification kit, Promega, Madison, USA) and an automatic extractor (Maxwell MDx 16, Promega). RT-PCR was performed on GeneAmp 9700 using Hotstar Taq polymerase (Qiagen) as follows;15 minutes at 95°C for initial denaturation, then 45 cycles at 95°C for 30 seconds, 62°C for 30 seconds, and 72°C for 60 seconds, then 5 minutes at 72°C for final extension. 30 ng of cDNA synthesized from total RNA was used to analyze. All PCR products were sequenced on both strands using the same primers and BigDye Terminator v3.1 Cycle Sequencing kits and a 3730 DNA analyzer (Applied Biosystems). The specific fusion primers for *PICALM–BRAF* provided in S8 Table.

### Fluorescence *in situ* hybridization

To evaluate *ETV6* rearrangement, we performed FISH analysis on FFPE tumor tissues using the Vysis LSI ETV6 spectrum Orange/Green probe (Abbot Molecular, Illinois, USA). These commercially-available probes are designed as a dual-color probe where the two regions across the break-point. For microscopic evaluation, at least 100 intact and nonoverlapping cell nuclei were scored for the presence of a split signal using a Zeiss Axio Imager with appropriate filters. Pictures were captured using a digital microscope camera ProgRes MF (Jenoptik, Germany) and analyzed with the Isis software (MetaSystems, Germany). The signal pattern interpretation was as follows: interphase nucleus with two colocalized green/orange fusion signals identified normal chromosomes, while a separated orange and green signals and green/orange fusion signals indicated rearranged gene. The positive threshold was defined as more than 10% of signals split and/or isolated orange signal in 100 tumor cells.

### Immunohistochemistry

To verity *STRN–ALK*, IHC staining was performed on FFPE tissue sections that were 4 m thick using an automated immunostainer (Leica Microsystems, Milton Keynes, UK). Briefly, the slides were heated for 20 min at 100°C in Epitope retrieval solution, pH 9.0 (Leica Microsystems). The slides were then incubated with a monoclonal mouse anti-human ALK antibody (Novocastra, Newcastle Upon Tyne, UK) at a dilution of 1:25. This antibody was raised against a C-terminal portion of the tyrosine kinase domain of ALK and was intended for the qualitative identification of ALK molecules in paraffin sections by light microscopy. Staining intensity was scored as 0 (no staining), 1+ (weak cytoplasmic staining without any background staining), and 2+ (strong cytoplasmic staining). Tumors with 1+ or 2+ expression in more than 10% of the tumor cells were deemed positive for ALK protein expression. For ALK IHC-positive cases, we subsequently performed IHC using an antibody against phosphorylated ALK (phosphor Y1507, Abcam, Cambridge, MA, USA) at a dilution of 1:100.

### Gene expression profiling and differentially expressed gene analysis

According to Ensembl gene set, we counted the number of reads aligned to each gene using HTSeq-count and normalized them via regularized log (rlog) transformation method of DESeq2 [65,66]. In this study, DEGs were determined by DESeq2 to have *q*-value < 0.05, |Log_2_ (fold change)| ≥ 1, and baseMean ≥ 100. The calculated *p*-values were adjusted to *q*-values for multiple testing using the Benjamini–Hochberg correction. The normalized gene expression values were applied to PCA using the most variable 500 genes. For heatmap display, the centered rlog values were applied to the K-means clustering algorithm using cluster 3.0 [67]. To identify pathways that were significantly enriched in DEGs, we applied them to the Molecular Signatures Database 5.0 [30].

### Thyroid differentiation score and ERK signature

As described by TCGA study, we calculated TDS using 16 thyroid metabolism and function genes. The rlog values from DESeq2 were first median-centered across 180 tumor samples, and then average values across the 16 genes in each tumor were determined as TDS.

TDS=Mean of median-centered rlog across16genes

To numerically represent activation level of MAPK signaling pathway, we implemented and modified ERK score calculation from TCGA. We applied identical method that was described in TDS calculation using 52 MAPK signaling pathway genes [68].

ERK score=Mean of median-centered rlog across52genes

### Jointly regulated block analysis

For JRB analysis, we selected genes in autosomes that have average FPKM ≥ 1.5 and were classified as protein-coding gene in Ensembl database. After that, we sorted genes by chromosomal coordinate and applied three normalization steps as follows: 1) Log (FPKM) of gene (gene A) in i^th^ tumor sample was *Z*-score transformed:

$$Z_{i,A}=\frac{Log\;(FPKM)-\mu }{\sigma }$$

where *μ* and *σ* represent average and standard deviation of Log (FPKM) of 81 normal tissues.

2) *Z*-score of gene (gene A) in i^th^ tumor sample was *Z*-score transformed:

$$Z_{Z,i,A}=\frac{Z_{i,A}-\mu_{i}}{\sigma_{i}}$$

where *μ*_*i*_ and *σ*_*i*_ represent average and standard deviation of *Z*-score of i^th^ tumor sample.

3) median *Z*-score of each chromosome arm of i^th^ tumor sample was median-centered by subtracting the median *Z*-score of all chromosome arms. After normalization steps, we defined arm with median-centered *Z*-score ≥ 0.5 and ≤ -0.5 as overexpressed and underexpressed JRB, respectively.

### Statistical analyses of clinical data

All statistical analyses were performed using SPSS version 20.0 (IBM Co, Armonk, NY, USA). Data are presented either as frequencies (%) or as mean ± standard deviation. Comparisons of categorical variables were performed using either the Pearson’s χ^2^ or Fisher’s exact test (if the number was < 5), and the independent t-test was used for continuous variables. Adjusted *p*-values for age and sex were obtained by the binomial or multinomial logistic regression analyses for categorical variables and by either the linear regression or analysis of covariance (ANCOVA) for continuous variables. A post-hoc Bonferroni test were used to determine which groups have statistically different proportion of clinical risk factors. Statistical significance was defined as two-sided *p-*values < 0.05.

### Data availability

We submitted all the sequenced paired-end reads to EBI European Nucleotide Archive database with accession number PRJEB11591 (Direct access: http://www.ebi.ac.uk/ena/data/view/PRJEB11591).

## Supporting Information

S1 Fig The driver candidates of thyroid tumors. (A) The amino acid coordinate of Ribonuclease III domain and distribution of mutations in *DICER1*. (B) The expression level of *DICER1* in normal, tumors with other mutations, and tumors with *DICER1* mutations. (C) The distribution of mutation across TCGA cases. Each column represents individual specimen. Right matrix was omitted due to there is no overlapped mutation. (D) The amino acid coordinate and distribution of mutations of *EIF1AX*.
(PDF)

S2 Fig Validation of driver candidates. (A) PCR and Sanger sequencing results of mutations in driver candidates. Upper and lower panel represent results of matched normal and tumor tissues, respectively. Left and right panel represent results of forward and reverse sequences, respectively. (B) RT-PCR and Sanger sequencing result of novel *PICALM*–*BRAF* fusion gene.
(PDF)

S3 Fig Supporting data on fusion genes. (A) Aberrant overexpression of fusion genes. (B) FISH result of *ETV6*–*NTRK3*. The separated green and orange signals and green/orange fusion signals indicate rearranged gene. (C) Photomicrograph image of ALK IHC result. IHC showed strong cytoplasmic staining in periphery of tumor.
(PDF)

S4 Fig The effect of LT and gene set usage on gene expression analysis. The result of K-means clustering via PCA on (A) All normal and tumor tissues using Ensembl gene set (Marked as histological subtype and driver gene). (B) All normal and tumor tissues using Ensembl gene set (Marked as LT and driver gene). (C) All tumors using Ensembl gene set (Marked as histological subtype and driver gene). (D) All tumors using Ensembl gene set (Marked as LT and driver gene). (E) Whole TCGA dataset using UCSC gene set. (F) Partial TCGA dataset (Two *DICER1*, four *EIF1AX*, four *PAX8–PPARG*, 15 *H/K/NRAS*, and 20 *BRAF* mutated samples were included) using UCSC gene set. Each cluster was represented by a 95.00% confidence ellipse.
(PDF)

S5 Fig The top 20 most significantly enriched KEGG pathways of upregulated DEGs in each molecular subtype. The significantly enriched KEGG pathways are marked by–log (*q*-value).
(PDF)

S6 Fig The top 20 most significantly enriched KEGG pathways of downregulated DEGs in each molecular subtype. The significantly enriched KEGG pathways are marked by–log (*q*-value).
(PDF)

S1 Table The list of selected fusion gene mutations identified from 180 study subjects. (XLSX)

S2 Table The result of gene set enrichment analysis using up-regulated genes in *ESRRA*-overexpression tumors. (XLSX)

S3 Table Gene set enrichment analysis on moderately downregulated genes in *H/K/NRAS* mutation with 22q deletion. (XLSX)

S4 Table Comparison of clinical risk factors among *H/K/NRAS* (+) with/without arm-level deletion of chromosome 22 groups (n = 39). (XLSX)

S5 Table Comparison of clinical risk factors among *BRAF*^V600E^ (+) with/without amplification of chromosome 18p groups (n = 67). (XLSX)

S6 Table Clinical information of 180 patients. (XLSX)

S7 Table RNA sequencing throughput of all study subjects. (XLSX)

S8 Table The primer sequences used in this study. (XLSX)
