# Supplementary material for: Comprehensive Analysis of the Transcriptional and Mutational Landscape of Follicular and Papillary Thyroid Cancers
Source: PLoS Genet. 2016 Aug 5;12(8):e1006239. doi: 10.1371/journal.pgen.1006239 (PMC4975456; doi:10.1371/journal.pgen.1006239)
Supplement: S1 Fig — (A) The amino acid coordinate of Ribonuclease III domain and distribution of mutations in DICER1. (B) The expression level of DICER1 in normal, tumors with other mutations, and tumors with DICER1 mutations. (C) The distribution of mutation across TCGA cases. Each column represents individual specimen. Right matrix was omitted due to there is no overlapped mutation. (D) The amino acid coordinate and distribution of mutations of EIF1AX. (PDF) [file pgen.1006239.s001.pdf]

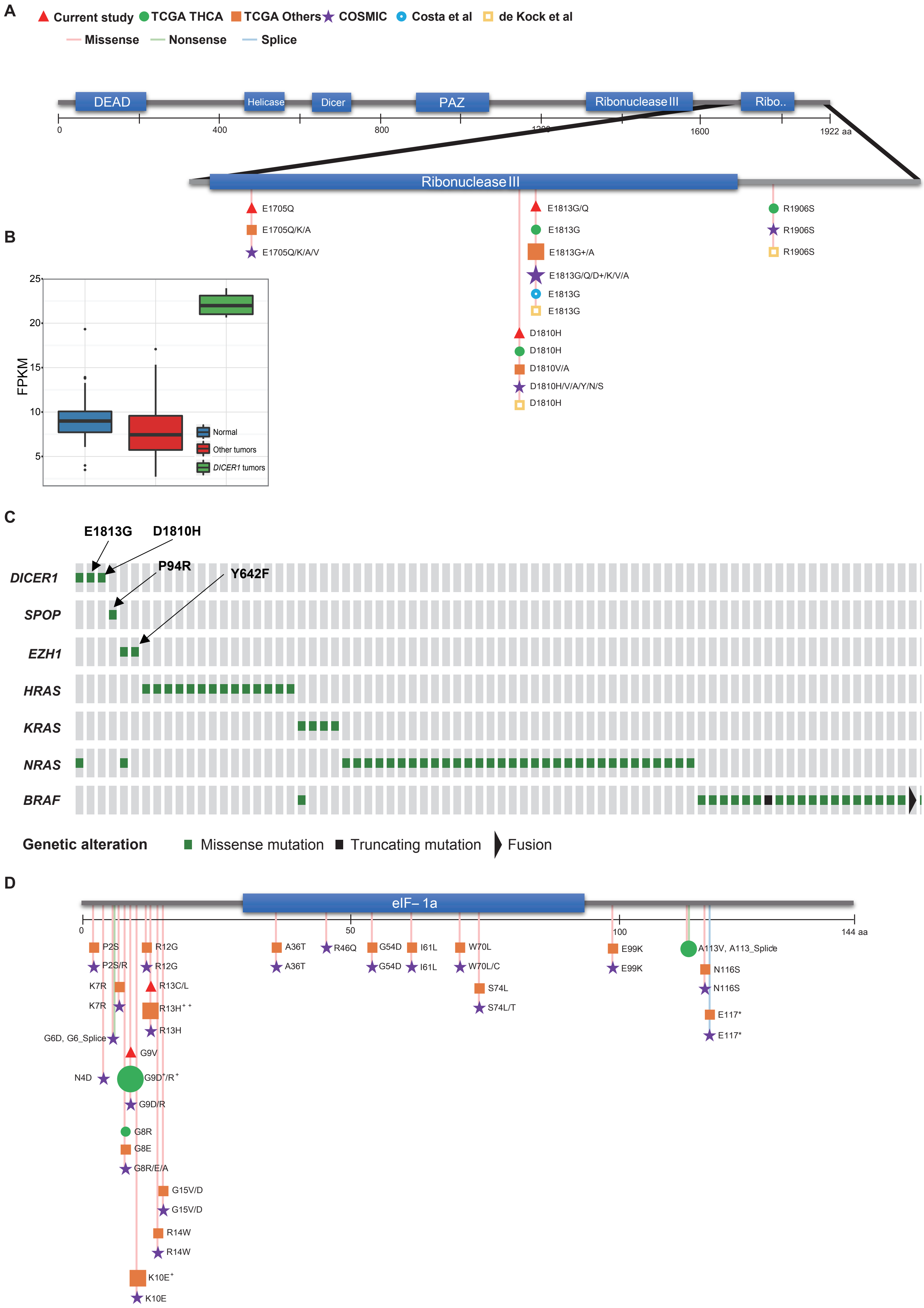
A
Current study
TCGA THCA
TCGA Others
COSMIC
Costa et al
de Kock et al
Missense
Nonsense
Splice
DEAD
Helicase
Dicer
PAZ
Ribonuclease III
Ribo..
0
400
800
1200
1600
1922 aa
Ribonuclease III
E1705Q
E1705Q/K/A
E1705Q/K/A/V
E1813G/Q
E1813G
E1813G+/A
E1813G/Q/D+/K/V/A
E1813G
E1813G
D1810H
D1810H
D1810V/A
D1810H/V/A/Y/N/S
D1810H
R1906S
R1906S
R1906S
B
FPKM
25
20
15
10
5
Normal
Other tumors
DICER1 tumors
C
E1813G
D1810H
P94R
Y642F
DICER1
SPOP
EZH1
HRAS
KRAS
NRAS
BRAF
Genetic alteration
Missense mutation
Truncating mutation
Fusion
D
eIF-1a
0
50
100
144 aa
P2S
P2S/R
K7R
K7R
G6D, G6_Splice
N4D
R12G
R12G
R13C/L
R13H++
R13H
G9V
G9D+/R+
G9D/R
G8R
G8E
G8R/E/A
G15V/D
G15V/D
R14W
R14W
K10E+
K10E
A36T
A36T
R46Q
G54D
G54D
I61L
I61L
W70L
W70L/C
S74L
S74L/T
E99K
E99K
A113V, A113_Splice
N116S
N116S
E117*
E117*
