# Supplementary material for: Comprehensive Analysis of the Transcriptional and Mutational Landscape of Follicular and Papillary Thyroid Cancers
Source: PLoS Genet. 2016 Aug 5;12(8):e1006239. doi: 10.1371/journal.pgen.1006239 (PMC4975456; doi:10.1371/journal.pgen.1006239)
Supplement: S2 Fig — (A) PCR and Sanger sequencing results of mutations in driver candidates. Upper and lower panel represent results of matched normal and tumor tissues, respectively. Left and right panel represent results of forward and reverse sequences, respectively. (B) RT-PCR and Sanger sequencing result of novel PICALM–BRAF fusion gene. (PDF) [file pgen.1006239.s002.pdf]

A

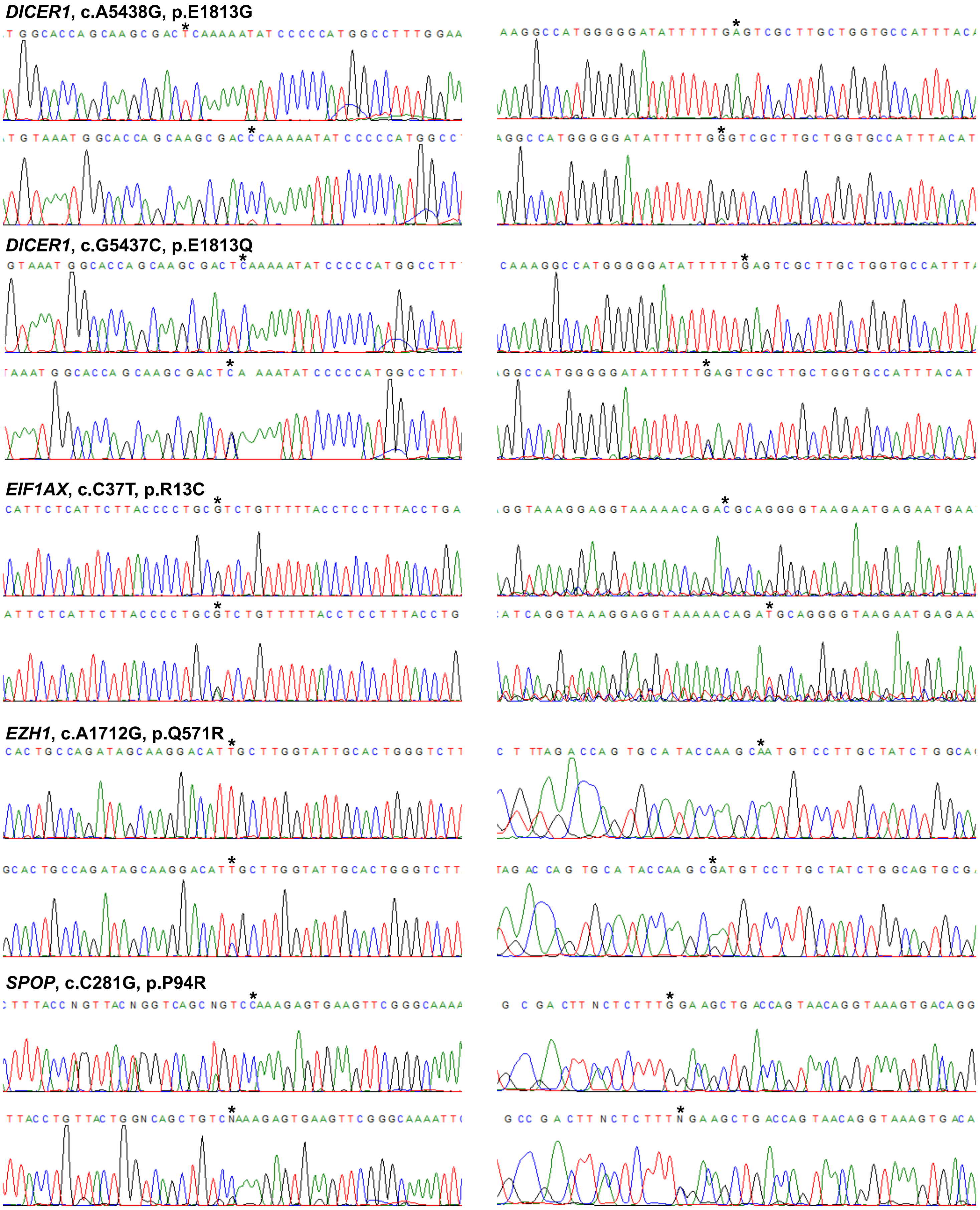
DICER1, c.A5438G, p.E1813G
DICER1, c.G5437C, p.E1813Q
EIF1AX, c.C37T, p.R13C
EZH1, c.A1712G, p.Q571R
SPOP, c.C281G, p.P94R

B

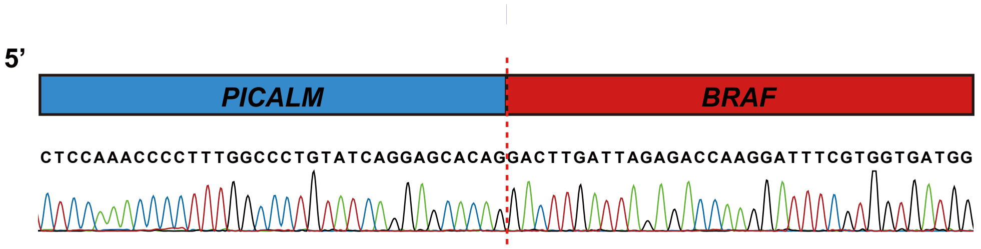
5'
3'
PICALM
BRAF
CTCCAAACCCCTTTGGCCCTGTATCAGGAGCACAGGACTTGATTAGAGACCAAGGATTTCGTGGTGATGG
