# Supplementary figures and images for: Comprehensive Analysis of the Transcriptional and Mutational Landscape of Follicular and Papillary Thyroid Cancers
Source: PLoS Genet. 2016 Aug 5;12(8):e1006239. doi: 10.1371/journal.pgen.1006239 (PMC4975456; doi:10.1371/journal.pgen.1006239)

**A**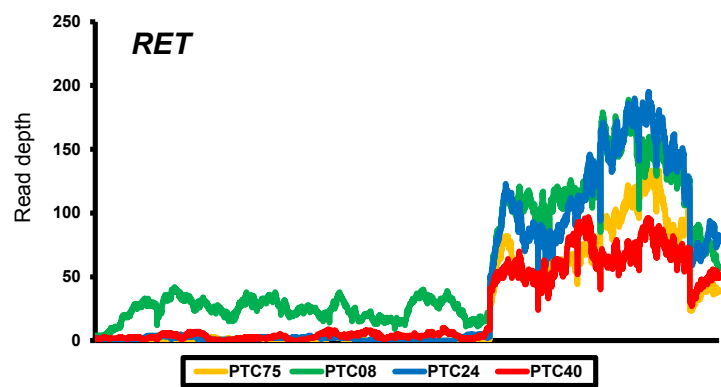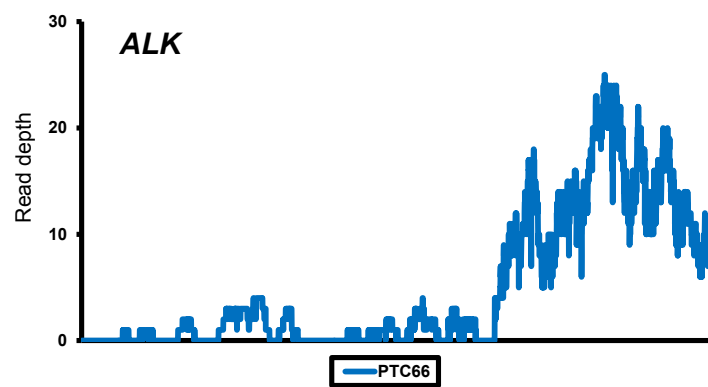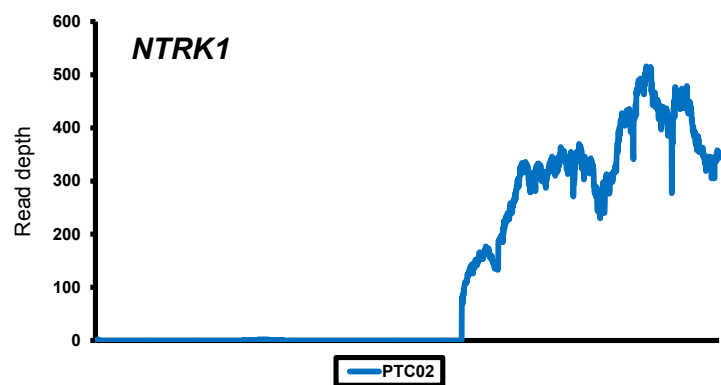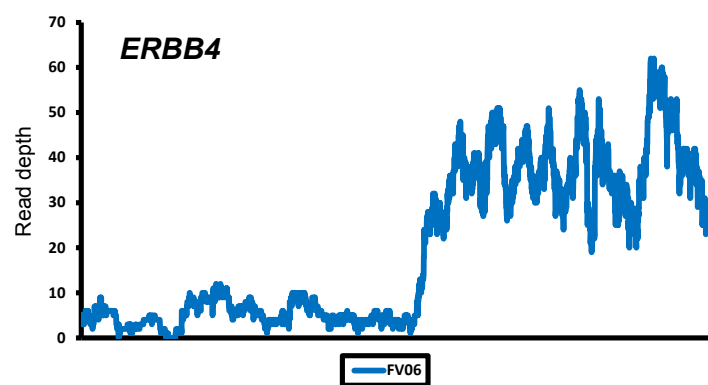**B**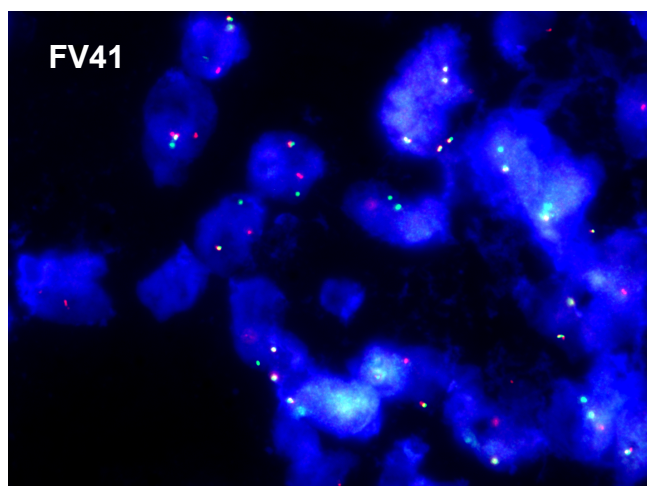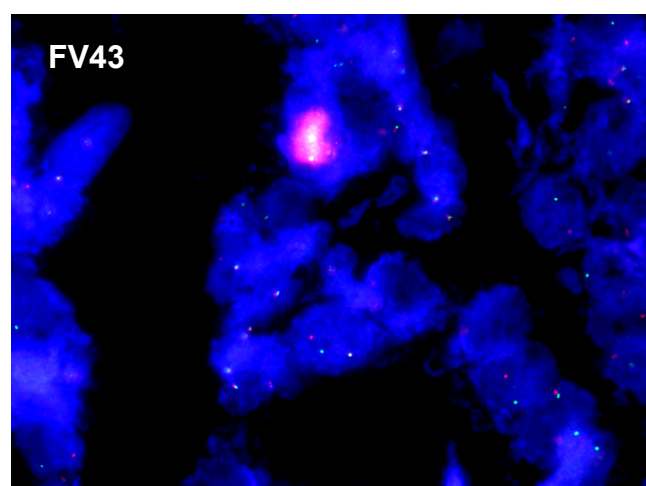**C**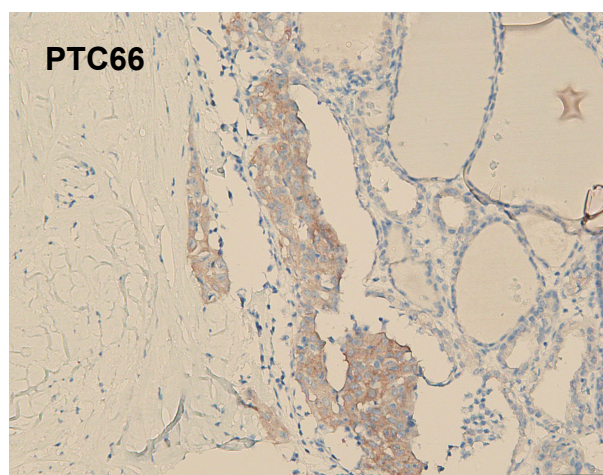

Supplement: S3 Fig — (A) Aberrant overexpression of fusion genes. (B) FISH result of ETV6–NTRK3. The separated green and orange signals and green/orange fusion signals indicate rearranged gene. (C) Photomicrograph image of ALK IHC result. IHC showed strong cytoplasmic staining in periphery of tumor. (PDF) [file pgen.1006239.s003.pdf]

A

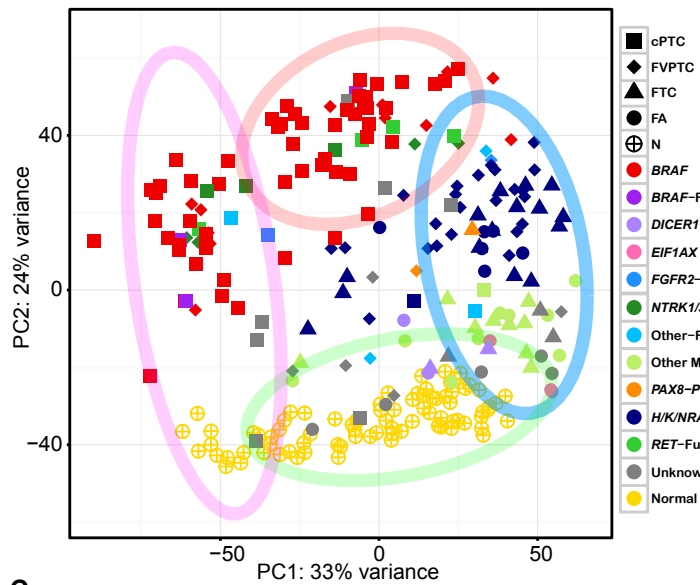

B

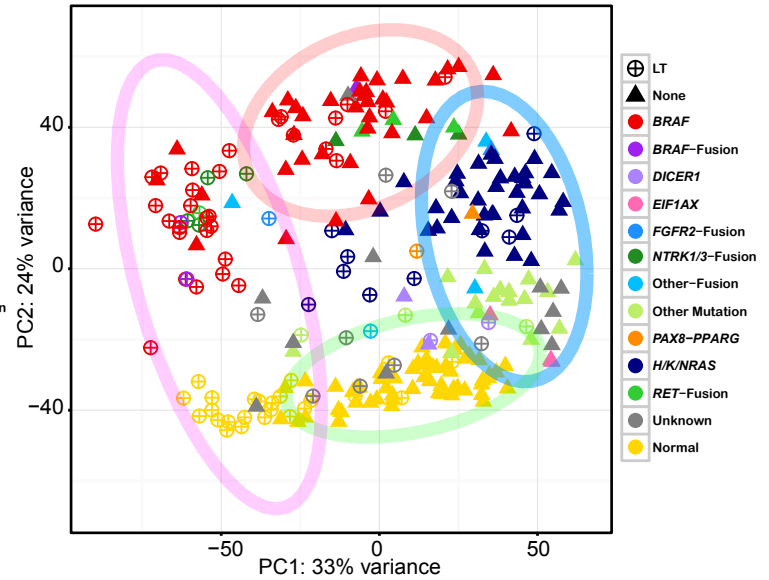

C

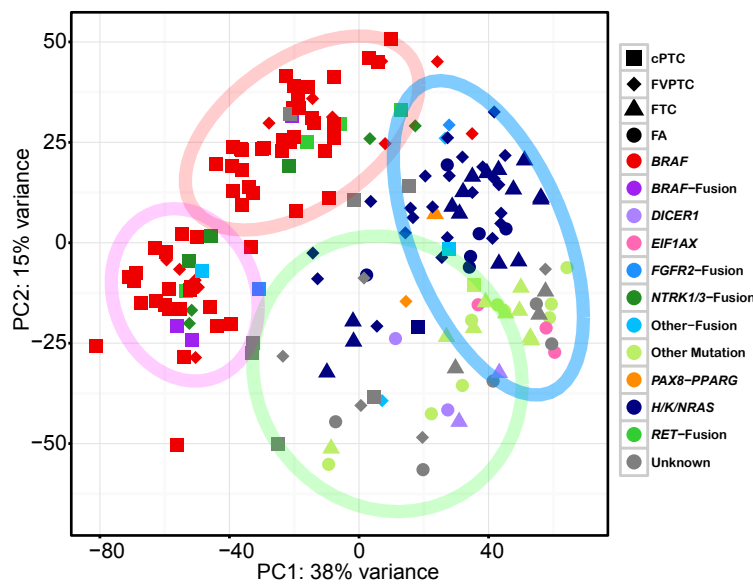

D

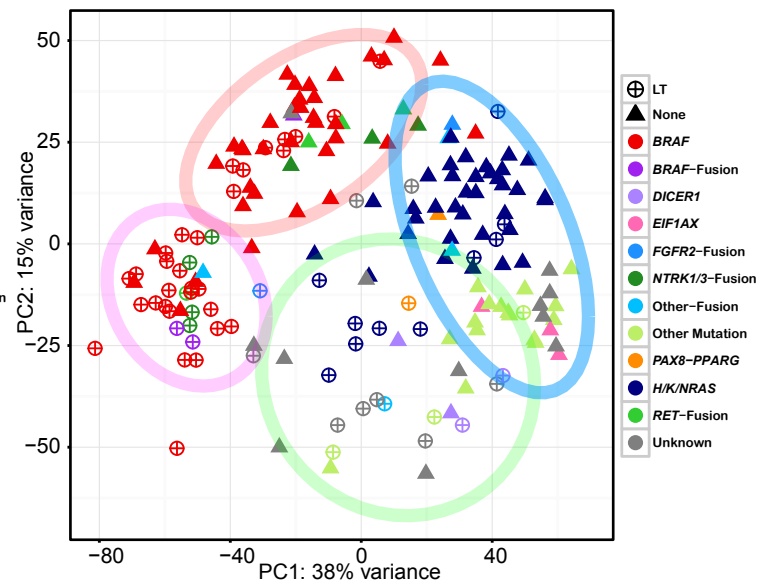

E

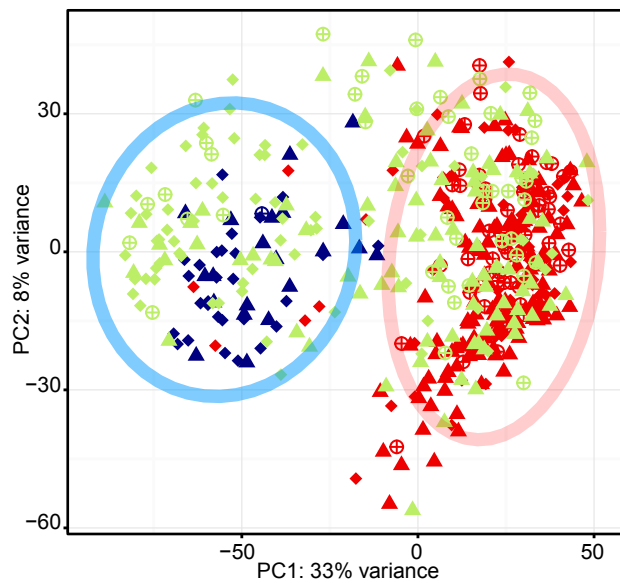

F

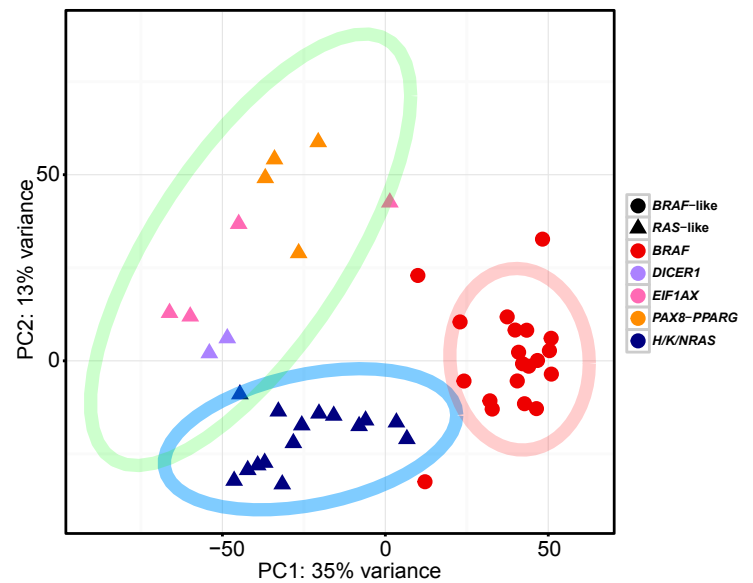

Supplement: S4 Fig — The result of K-means clustering via PCA on (A) All normal and tumor tissues using Ensembl gene set (Marked as histological subtype and driver gene). (B) All normal and tumor tissues using Ensembl gene set (Marked as LT and driver gene). (C) All tumors using Ensembl gene set (Marked as histological subtype and driver gene). (D) All tumors using Ensembl gene set (Marked as LT and driver gene). (E) Whole TCGA dataset using UCSC gene set. (F) Partial TCGA dataset (Two DICER1, four EIF1AX, four PAX8–PPARG, 15 H/K/NRAS, and 20 BRAF mutated samples were included) using UCSC gene set. Each cluster was represented by a 95.00% confidence ellipse. (PDF) [file pgen.1006239.s004.pdf]
