# Supplementary material for: Comprehensive Analysis of the Transcriptional and Mutational Landscape of Follicular and Papillary Thyroid Cancers
Source: PLoS Genet. 2016 Aug 5;12(8):e1006239. doi: 10.1371/journal.pgen.1006239 (PMC4975456; doi:10.1371/journal.pgen.1006239)

Category		KEGG pathway
**Metabolism**	Global and overview maps	FATTY ACID METABOLISM
	Carbohydrate metabolism	CITRATE CYCLE TCA CYCLE
		PROPANOATE METABOLISM
		GLYCOLYSIS GLUCONEOGENESIS
		PYRUVATE METABOLISM
		GLYOXYLATE AND DICARBOXYLATE METABOLISM
		BUTANOATE METABOLISM
	Energy metabolism	OXIDATIVE PHOSPHORYLATION
	Lipid metabolism	SPHINGOLIPID METABOLISM
	Nucleotide metabolism	PURINE METABOLISM
		PYRIMIDINE METABOLISM
	Amino acid metabolism	VALINE LEUCINE AND ISOLEUCINE DEGRADATION
		TRYPTOPHAN METABOLISM
		ARGININE AND PROLINE METABOLISM
		ALANINE ASPARTATE AND GLUTAMATE METABOLISM
	Metabolism of other amino acids	GLUTATHIONE METABOLISM
	Metabolism of cofactors and vitamins	PORPHYRIN AND CHLOROPHYLL METABOLISM
**Genetic Information Processing**	Transcription	RNA POLYMERASE
		SPLICEOSOME
	Translation	RIBOSOME
		AMINOACYL TRNA BIOSYNTHESIS
	Folding, sorting and degradation	PROTEASOME
**Environmental Information Processing**	Signal transduction	MAPK SIGNALING PATHWAY
		CALCIUM SIGNALING PATHWAY
		WNT SIGNALING PATHWAY
	Membrane transport	ABC TRANSPORTERS
	Signal transduction	PHOSPHATIDYLINOSITOL SIGNALING SYSTEM
		VEGF SIGNALING PATHWAY
		MTOR SIGNALING PATHWAY
	Signaling molecules and interaction	ECM RECEPTOR INTERACTION
		CELL ADHESION MOLECULES (CAMS)
		CYTOKINE CYTOKINE RECEPTOR INTERACTION
**Cellular Processes**	Transport and catabolism	LYSOSOME
		PEROXISOME
	Cell motility	REGULATION OF ACTIN CYTOSKELETON
		CELL CYCLE
	Cell growth and death	P53 SIGNALING PATHWAY
	Cellular community	FOCAL ADHESION
		TIGHT JUNCTION
		GAP JUNCTION
**Organismal Systems**	Immune system	COMPLEMENT AND COAGULATION CASCADES
		CHEMOKINE SIGNALING PATHWAY
		NATURAL KILLER CELL MEDIATED CYTOTOXICITY
	Endocrine system	INSULIN SIGNALING PATHWAY
		MELANOGENESIS
		GNRH SIGNALING PATHWAY
		PROGESTERONE MEDIATED OOCYTE MATURATION
		PPAR SIGNALING PATHWAY
		ADIPOCYTOKINE SIGNALING PATHWAY
	Circulatory system	CARDIAC MUSCLE CONTRACTION
		VASCULAR SMOOTH MUSCLE CONTRACTION
	Nervous system	LONG TERM DEPRESSION
		LONG TERM POTENTIATION
	Development	AXON GUIDANCE
Human Diseases	Cancers: Overview	PATHWAYS IN CANCER
	Cancers: Specific types	SMALL CELL LUNG CANCER
		MELANOMA
		CHRONIC MYELOID LEUKEMIA
		GLIOMA
		PANCREATIC CANCER
		ACUTE MYELOID LEUKEMIA
		PROSTATE CANCER
		BLADDER CANCER
		ENDOMETRIAL CANCER
		RENAL CELL CARCINOMA
	Immune diseases	SYSTEMIC LUPUS ERYTHEMATOSUS
		ASTHMA
	Neurodegenerative diseases	PARKINSONS DISEASE
		ALZHEIMERS DISEASE
		HUNTINGTONS DISEASE
	Cardiovascular diseases	ARVC
		VIRAL MYOCARDITIS
		DILATED CARDIOMYOPATHY
		HCM
	Endocrine and metabolic diseases	TYPE II DIABETES MELLITUS
	Infectious diseases: Parasitic	LEISHMANIA INFECTION

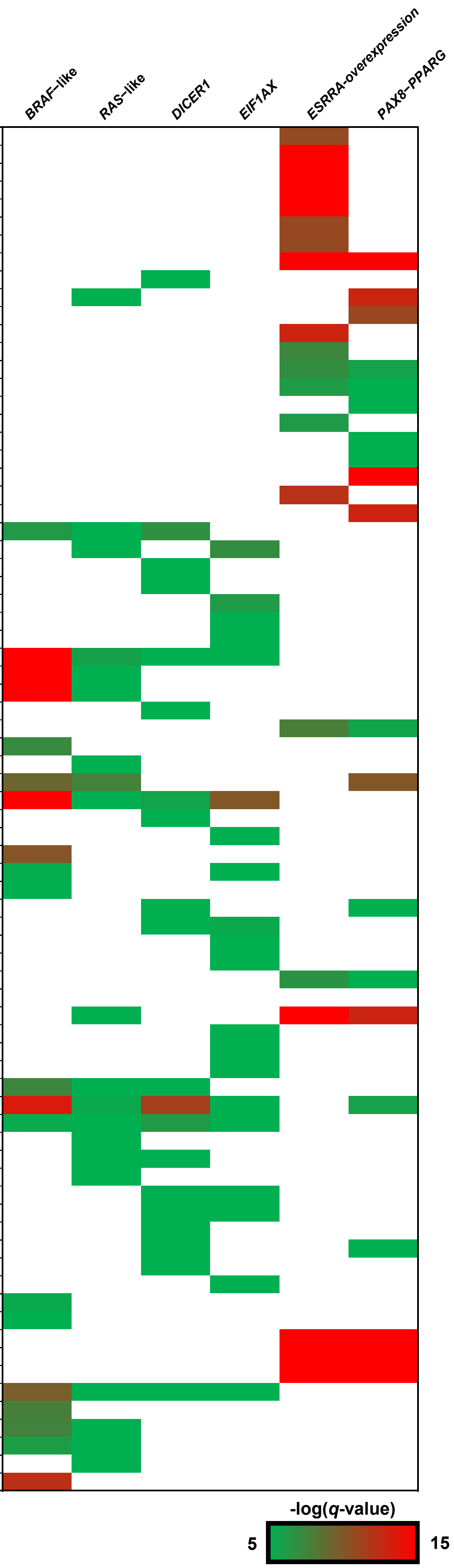

Supplement: S5 Fig — The significantly enriched KEGG pathways are marked by–log (q-value). (PDF) [file pgen.1006239.s005.pdf]
