# Supplementary material for: Comprehensive Analysis of the Transcriptional and Mutational Landscape of Follicular and Papillary Thyroid Cancers
Source: PLoS Genet. 2016 Aug 5;12(8):e1006239. doi: 10.1371/journal.pgen.1006239 (PMC4975456; doi:10.1371/journal.pgen.1006239)

| Category | | KEGG pathway |
|---|---|---|
| **Metabolism** | Lipid metabolism | GLYCEROLIPID METABOLISM |
| | | STEROID HORMONE BIOSYNTHESIS |
| | | ARACHIDONIC ACID METABOLISM |
| | Amino acid metabolism | TRYPTOPHAN METABOLISM |
| | | ARGININE AND PROLINE METABOLISM |
| | | GLYCINE SERINE AND THREONINE METABOLISM |
| | | TYROSINE METABOLISM |
| | Xenobiotics biodegradation and metabolism | METABOLISM OF XENOBIOTICS BY CYTOCHROME P450 |
| | | DRUG METABOLISM CYTOCHROME P450 |
| **Environmental Information Processing** | Signal transduction | MAPK SIGNALING PATHWAY |
| | | CALCIUM SIGNALING PATHWAY |
| | | WNT SIGNALING PATHWAY |
| | Membrane transport | ABC TRANSPORTERS |
| | | VEGF SIGNALING PATHWAY |
| | | HEDGEHOG SIGNALING PATHWAY |
| | | TGF BETA SIGNALING PATHWAY |
| | | JAK STAT SIGNALING PATHWAY |
| | Signaling molecules and interaction | ECM RECEPTOR INTERACTION |
| | | CELL ADHESION MOLECULES (CAMS) |
| | | CYTOKINE CYTOKINE RECEPTOR INTERACTION |
| **Cellular process** | Cell motility | REGULATION OF ACTIN CYTOSKELETON |
| | Cellular community | FOCAL ADHESION |
| | | TIGHT JUNCTION |
| **Organismal Systems** | Immune system | COMPLEMENT AND COAGULATION CASCADES |
| | | CHEMOKINE SIGNALING PATHWAY |
| | | NATURAL KILLER CELL MEDIATED CYTOTOXICITY |
| | | B CELL RECEPTOR SIGNALING PATHWAY |
| | | T CELL RECEPTOR SIGNALING PATHWAY |
| | | HEMATOPOIETIC CELL LINEAGE |
| | | LEUKOCYTE TRANSENDOTHELIAL MIGRATATION |
| | | FC GAMMA R MEDIATED PHAGOCYTOSIS |
| | | ANTIGEN PROCESSING AND PRESENTATION |
| | | INTESTINAL IMMUNE NETWORK FOR IGA PRODUCTION |
| | | NATURAL KILLER CELL MEDIATED CYTOXICITY |
| | | TOLL LIKE RECEPTOR SIGNALING PATHWAY |
| | Endocrine system | MELANOGENESIS |
| | | ADIPOCYTOKINE SIGNALING PATHWAY |
| | Circulatory system | VASCULAR SMOOTH MUSCLE CONTRACTION |
| | Excretory system | ALDOSTERONE REGULATED SODIUM REABSORPTION |
| | | PROXIMAL TUBULE BICARBONATE RECLAMATION |
| | Development | AXON GUIDANCE |
| Human Diseases | Cancers: Overview | PATHWAYS IN CANCER |
| | Cancers: Specific types | ACUTE MYELOID LEUKEMIA |
| | | BASAL CELL CARCINOMA |
| | | COLORECTAL CANCER |
| | Immune diseases | SYSTEMIC LUPUS ERYTHEMATOSUS |
| | | ASTHMA |
| | | PRIMARY IMMUNODEFICIENCY |
| | | ALLOGRAFT REJECTION |
| | | GRAFT VERSUS HOST DISEASE |
| | | AUTOIMMUNE THYROID DISEASE |
| | Cardiovascular diseases | VIRAL MYOCARDITIS |
| | | DILATED CARDIOMYOPATHY |
| | | HCM |
| | Endocrine and metabolic diseases | TYPE I DIABETES MELLITUS |
| | Infectious diseases: Parasitic | LEISHMANIA INFECTION |

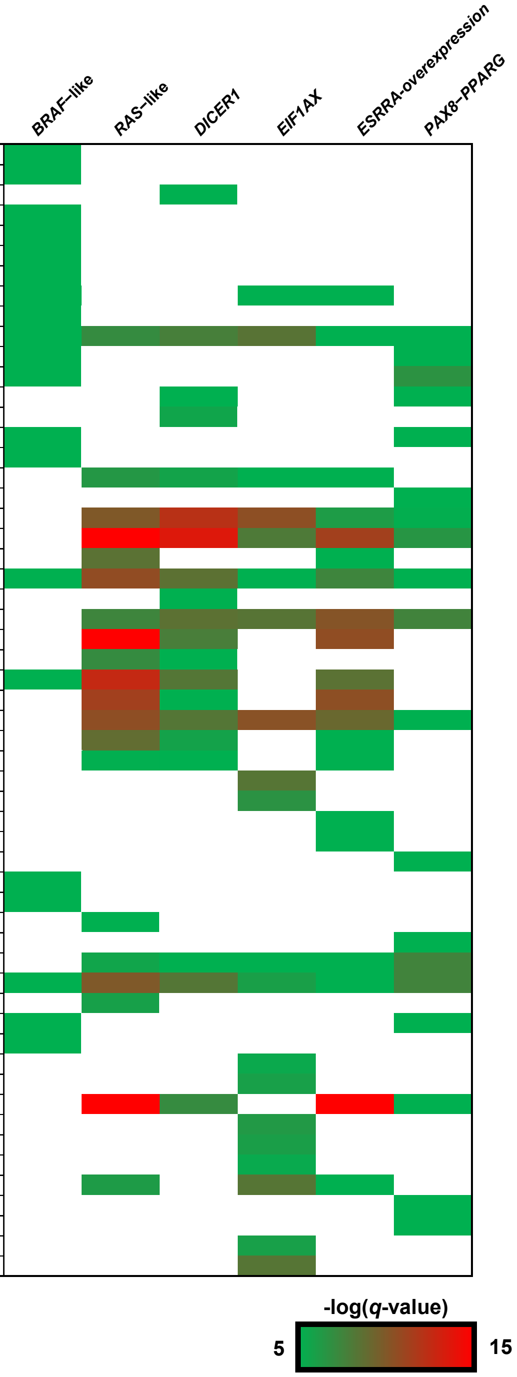

Supplement: S6 Fig — The significantly enriched KEGG pathways are marked by–log (q-value). (PDF) [file pgen.1006239.s006.pdf]
